# Role of progesterone action in inguinal hernia formation via skeletal muscle fibrosis and atrophy

**DOI:** 10.1172/jci.insight.193208

**Published:** 2025-06-12

**Authors:** Tianming You, Mehrdad Zandigohar, Tanvi Potluri, Natalie Piehl, John S. Coon V, Elizabeth Baker, Maya Kafali, Yang Dai, Jonah J. Stulberg, David J. Escobar, Richard L. Lieber, Hong Zhao, Serdar E. Bulun

**Affiliations:** 1 Department of Obstetrics & Gynecology, Feinberg School of Medicine, Northwestern University, Chicago, Illinois, USA.; 2 Department of Biomedical Engineering, University of Illinois Chicago, Chicago, Illinois, USA.; 3 Department of Surgery, McGovern Medical School at the University of Texas Health Sciences Center, Houston, Texas, USA.; 4 Department of Pathology, Feinberg School of Medicine, and; 5 Departments of Physical Medicine and Rehabilitation and Biomedical Engineering, Northwestern University, Chicago, Illinois, USA.; 6 Research Service, Hines VA Medical Center, Maywood, Illinois, USA.; 7 Shirley Ryan AbilityLab, Chicago, Illinois, USA.

**Keywords:** Cell biology, Endocrinology, Muscle biology, Fibrosis, Sex hormones, Skeletal muscle

## Abstract

More than 1 in 4 men will undergo surgery for inguinal hernia, which is commonly associated with fibrotic degeneration of the lower abdominal muscle (LAM) in the groin region. Utilizing a male mouse model expressing the human aromatase gene (*Arom^hum^*), previous studies showed that locally produced estradiol acting via estrogen receptor α in LAM fibroblasts leads to fibrosis, myofiber atrophy, and hernia development. Here, we found that upregulation of progesterone receptor (PGR) in a LAM fibroblast population mediates this estrogenic effect. A PGR-selective progesterone antagonist in *Arom^hum^* mice decreased LAM fibrosis and atrophy, preventing hernia formation and stopping progression of existing hernias. Addition of progesterone to estradiol treatment was essential for early-onset development of LAM fibrosis and large hernias in wild-type mice, which was averted by a progesterone antagonist. Single-nuclei multiomics sequencing of herniated LAM revealed a unique population of *Pgr*-expressing fibroblasts that promotes fibrosis and myofiber atrophy through TGF-β2 signaling. Multiomics findings were validated in vivo in herniated LAM tissues of both mice and adult men. Our findings suggest an important and rare pathologic role of progesterone signaling in males and provide evidence for progesterone antagonists as a nonsurgical alternative for inguinal hernia management.

## Abstract 2

Utilizing mouse models and muscle samples from hernia patients, we show the potential for progesterone antagonists as a pharmacologic treatment for inguinal hernias.

## Introduction

Approximately 27% of men and 3% of women develop inguinal hernias in their lifetime (1). Inguinal hernia repair surgery remains one of the most common procedures performed in the United States (~1,000,000/year) and around the world, and annual health care costs directly attributable to inguinal hernias exceed $2.5 billion (2–5). Although surgery can resolve most inguinal hernias in otherwise healthy patients, recurrent hernias are more likely to occur in older men or those with significant comorbidities (6–10). These refractory cases continue to challenge surgeons and patients due to high rates of morbidity, mortality, and complications (e.g., infection and chronic pain). However, there is a significant gap in our understanding of the etiology and pathophysiology of inguinal hernia.

The lower abdominal muscle (LAM) tissue, where inguinal hernias occur, is comprised of layers of skeletal muscle myofibers surrounded by stromal tissue, a mixture of fibroblasts and extracellular matrix (ECM). Hernia formation is accompanied by weakening of the LAM tissue via fibrosis and myofiber atrophy (11). Our lab created a humanized aromatase mouse model (*Arom^hum^*) that recapitulates this phenotype in the LAM and highlights the importance of estradiol (E2) action in LAM fibrosis and atrophy (12). *Arom^hum^* mice, which express the human aromatase gene (*CYP19A1*) and its native promoters, mimic human aromatase activity across various tissues, including LAM, where it facilitates local conversion of testosterone to E2 (13–17). Aromatase is not normally expressed in the abdominal muscle of wild-type (WT) mice. Expression of aromatase in the LAM significantly increased LAM E2 to levels comparable to humans and resulted in tissue fibrosis and scrotal hernia formation in 100% of male *Arom^hum^* mice (12, 18).

Histological analysis of *Arom^hum^* LAM tissue revealed significantly increased stromal expression of estrogen receptor α (ESR1) compared with WT LAM tissue (12). Additionally, single-cell RNA sequencing (RNA-seq) analysis of LAM tissue in WT and *Arom^hum^* mice revealed a group of fibroblasts upregulated in *Arom^hum^* LAM with high levels of *Esr1* expression (19). These fibroblasts expressed several profibrotic genes characteristic of activated fibroblasts (19). Furthermore, both pharmacologic inhibition and genetic ablation of ESR1 in the LAM fibroblasts of male *Arom^hum^* mice completely prevented LAM fibrosis and hernia development, highlighting the importance of fibroblast-specific E2/ESR1 signaling in causing hernias (20). However, the pathways and downstream mechanisms of E2/ESR1 signaling that cause fibroblast activation and LAM fibrosis remain unknown.

Progesterone receptor (PGR) is a well-established target gene of E2/ESR1 signaling, with activation of ESR1 leading to increased expression of PGR in the breast, ovary, and uterine endometrium (21–23). Activation of PGR signaling by progesterone (P4) has been shown to contribute to fibrosis in the lung and peritoneum (24, 25). P4/PGR signaling has also been shown to increase cell proliferation and ECM deposition in uterine leiomyoma (26). However, the role of P4/PGR signaling in skeletal muscle is poorly studied, particularly in males. In this study, we found that P4/PGR signaling in a unique population of LAM fibroblasts plays a central role in LAM fibrosis and that pharmacologic inhibition of P4/PGR signaling prevents hernia development and stops hernia enlargement. We employed single-nuclei multiomic sequencing (snRNA-seq and single-nuclei assay for transposase-accessible chromatin using sequencing [snATAC-seq]) and Xenium spatial transcriptomics in mouse LAM tissue to provide evidence that P4/PGR potentially mediates LAM fibrosis and myofiber atrophy through transforming growth factor β (TGF-β) signaling. Lastly, we showed that key mediators of these signaling pathways are present in the LAM of male hernia patients. These findings highlight the potential of PGR antagonists as a nonsurgical alternative to inguinal hernia management and introduce an important pathologic role for P4/PGR signaling in men.

## Results

### Effect of E2/ESR1-induced PGR expression on LAM fibroblast proliferation in Arom^hum^ mice and human hernia tissue.

**Figure 1:**
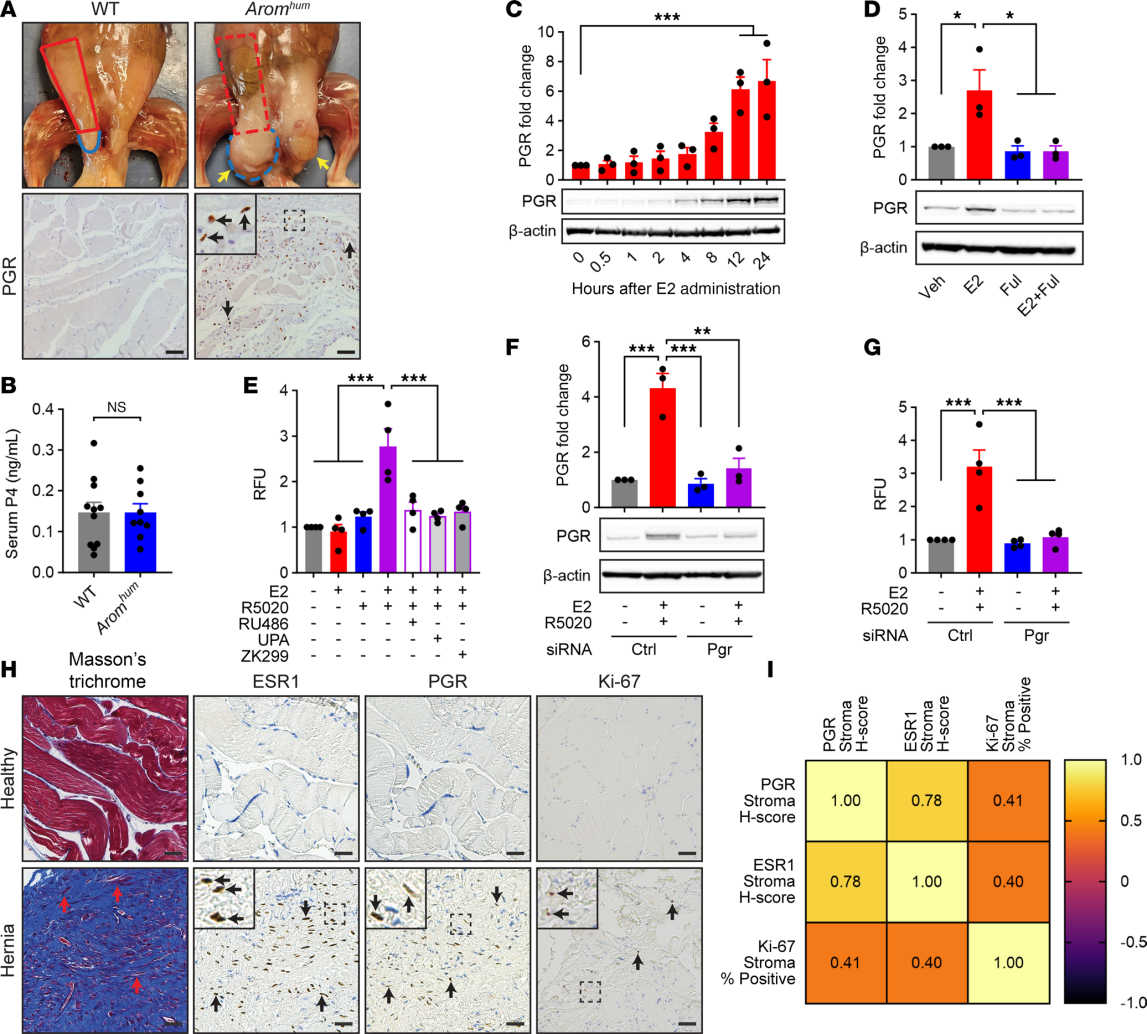
Effect of E2/ESR1-induced PGR expression on LAM fibroblast proliferation in *Arom^hum^* mice and human hernia tissue. (**A**) Representative hernia images (yellow arrows) and IHC staining for PGR expression in the LAM of WT and *Arom^hum^* mice. Normal extent of LAM (solid red) and scrotum (solid blue), as well as extent of herniated LAM (dotted red) and scrotum (dotted blue) are highlighted. (**B**) Serum progesterone (P4) levels of WT and *Arom^hum^* mice (*n* = 9–11/group, mean ± SEM, *t* test). (**C** and **D**) Immunoblot analysis of PGR expression in primary *Arom^hum^* LAM fibroblasts (**C**) over 24-hour time course after E2 treatment and (**D**) after administration of E2 and/or fulvestrant (Ful) (*n* = 3/group, mean ± SEM, 1-way ANOVA). (**E**) EdU analysis of cell proliferation in primary *Arom^hum^* LAM fibroblasts after administration of E2, R5020, and/or various progesterone antagonists (mifepristone [RU486], ulipristal acetate [UPA], onapristone [ZK299]) (RFU = relative fluorescence units; *n* = 4/group, mean ± SEM, 1-way ANOVA). (**F**) Immunoblot analysis of PGR expression in primary *Arom^hum^* LAM fibroblasts administration of nontargeting (Ctrl) or Pgr siRNA (*n* = 3/group, mean ± SEM, 2-way ANOVA). (**G**) EdU analysis of cell proliferation in primary *Arom^hum^* LAM fibroblasts after administration of E2 and R5020 with or without nontargeting (Ctrl) or Pgr siRNA (*n* = 4/group, mean ± SEM, 2-way ANOVA). (**H**) Representative images of Masson’s trichrome staining and IHC staining for ESR1, PGR, and Ki-67 in human LAM tissues from inguinal hernia sites and adjacent healthy muscle tissues. Red arrows show atrophying myofibers in herniated LAM tissue. (**I**) Spearman’s (*r*_s_) correlation between PGR, ESR1, and Ki-67 scores (*n* = 43, *P* < 0.0001 for each comparison). Scale bars: 50 μm. *P < 0.05; **P < 0.01; ***P < 0.001.

We previously demonstrated that both local E2 and fibroblast ESR1 levels are significantly increased in the LAM tissue of *Arom^hum^* mice compared with WT mice (12, 19). Further analysis via immunohistochemistry (IHC) revealed the presence of PGR protein expression in the stroma of *Arom^hum^* LAM, but little to no expression in WT LAM (Figure 1A). Additionally, serum P4 levels in both WT and *Arom^hum^* mice were comparable to those found in human males (Figure 1B) (27).

To determine whether the increase in stromal LAM PGR expression is due to E2/ESR1 signaling in fibroblasts, primary fibroblasts from the LAM of *Arom^hum^* mice were isolated and cultured in the presence of E2 and/or fulvestrant, an E2/ESR1 antagonist. A 24-hour time-course treatment with E2 induced PGR protein expression in cultured LAM fibroblasts (Figure 1C). Administration of fulvestrant concurrently with E2 fully prevented this induction (Figure 1D).

To determine whether PGR signaling affects primary LAM fibroblasts, we treated LAM fibroblasts with both E2 and R5020, a synthetic progesterone analogue, and measured cell proliferation via incorporation of EdU into DNA. Simultaneous treatment with both E2 and R5020 significantly increased fibroblast proliferation, whereas neither E2 nor R5020 alone achieved the same effect (Figure 1E). Furthermore, concurrent administration of P4/PGR antagonists mifepristone (RU486), ulipristal acetate (UPA), or onapristone (ZK299) completely prevented E2/R5020-induced cell proliferation (Figure 1E). siRNA knockdown of PGR in LAM fibroblasts had a similar attenuating effect on E2/R5020-induced cell proliferation (Figure 1, F and G). Taken together, these results provide evidence for the importance of PGR signaling in the activation and proliferation of LAM fibroblasts of *Arom^hum^* mice.

We also sought to determine whether the positive correlation between ESR1 expression, PGR expression, and fibroblast proliferation was present in the abdominal muscle of human patients with inguinal hernias. LAM biopsies were obtained from 43 men undergoing hernia repair surgery (28–80 years of age). These biopsies included tissue directly from the herniated LAM and matched healthy LAM tissue adjacent to the hernia site. Regression analysis revealed a significant correlation between the expression of stromal ESR1 and PGR on IHC as well as a significant correlation between PGR expression and cell proliferation, as measured by stromal Ki-67 expression on IHC (Figure 1, H and I). These findings suggest that both the E2/ESR1-induced expression of PGR and the E2/P4-induced increase in cell proliferation seen in LAM fibroblasts of *Arom^hum^* mice may also occur in human tissue.

### Inhibition of P4/PGR signaling prevents hernia development and delays further hernia enlargement in Arom^hum^ mice.

**Figure 2:**
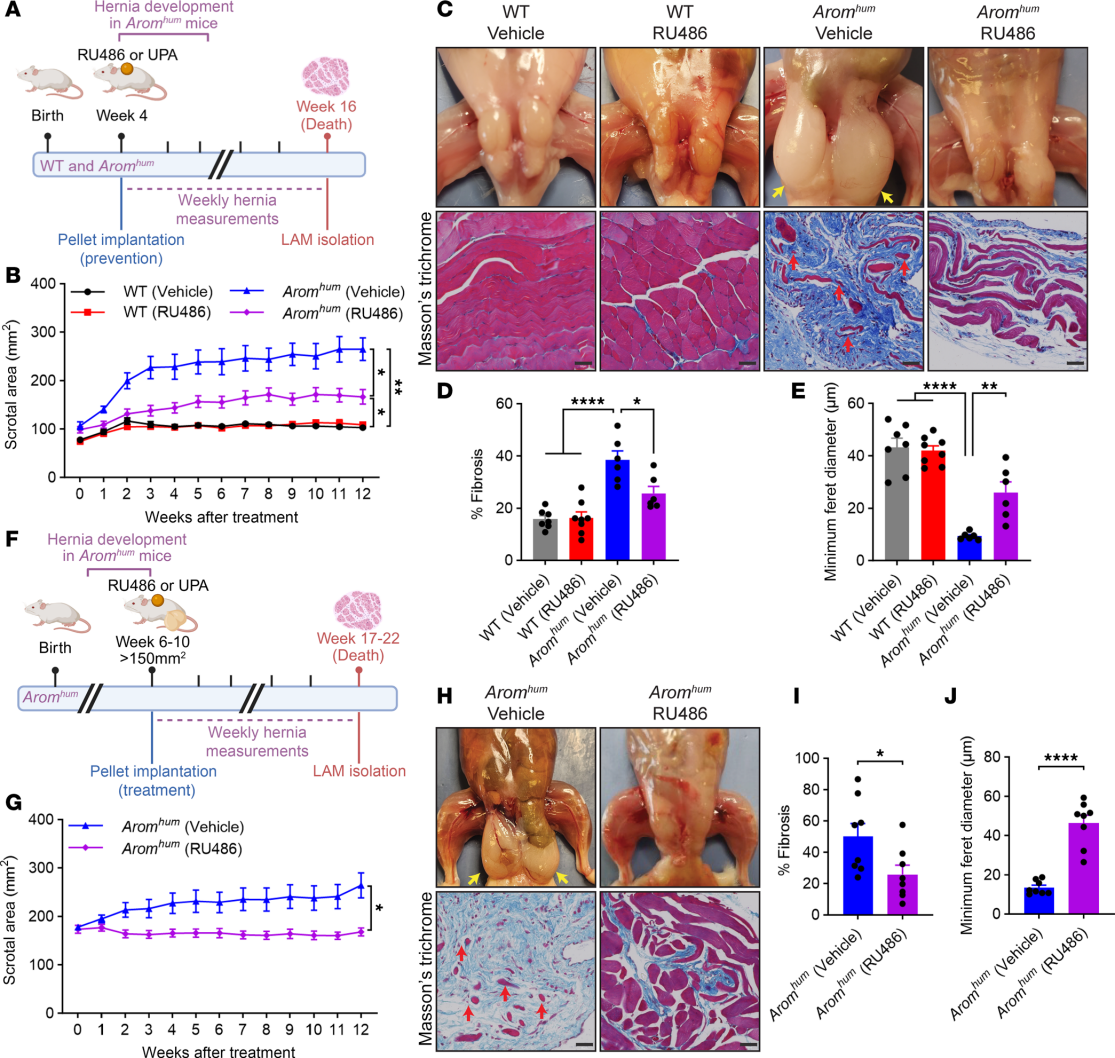
Treatment with progesterone antagonist RU486 prevents hernia development and delays further hernia enlargement in *Arom^hum^* mice. (**A**) Schematic for 12-week RU486 or UPA (Supplemental Figure 2) treatment in 4-week-old WT and *Arom^hum^* mice for the prevention of hernia development. Created with BioRender.com. (**B**) Scrotal/hernia area measurements of WT and *Arom^hum^* mice treated with RU486 as in **A** (*n* = 9–10/group, mean ± SEM, repeated-measures ANOVA). (**C**) Representative images of LAM morphology and Masson’s trichrome staining in WT and *Arom^hum^* mice after 12-week RU486 preventive treatment. Bilateral scrotal hernias (yellow arrows) and atrophying myofibers in herniated LAM tissue (red arrows) are highlighted for vehicle-treated *Arom^hum^* mice. (**D** and **E**) Quantification of (**D**) fibrotic area and (**E**) minimum Feret diameter in LAM tissues after 12-week RU486 preventive treatment (*n* = 6–8/group, mean ± SEM, 2-way ANOVA). (**F**) Schematic for 12-week RU486 or UPA (Supplemental Figure 2) treatment in 6- to 10-week-old *Arom^hum^* mice with established scrotal hernias. Created with BioRender.com. (**G**) Scrotal/hernia area measurements of *Arom^hum^* mice treated with RU486 as in **F** (*n* = 10–11/group, mean ± SEM, multiple *t* test). (**H**) Representative images of LAM morphology and Masson’s trichrome staining after 12 weeks of RU486 treatment of established hernias in *Arom^hum^* mice. Bilateral scrotal hernias (yellow arrows) and atrophying myofibers in herniated LAM tissue (red arrows) are highlighted for vehicle-treated *Arom^hum^* mice. (**I** and **J**) Quantification of (**I**) fibrotic area and (**J**) minimum Feret diameter in LAM tissues after 12-week RU486 treatment of established hernias in *Arom^hum^* mice (*n* = 8/group, mean ± SEM, *t* test). Scale bars: 50 μm. *P < 0.05; **P < 0.01; ****P < 0.0001.

To determine whether P4/PGR antagonists can prevent hernia development, we treated *Arom^hum^* mice and WT littermates with RU486 or UPA starting at 3–4 weeks of age, prior to hernia formation (Figure 2A). ZK299 was not used due to its hepatotoxicity (28). During the 12-week treatment period, vehicle-treated (Veh-treated) *Arom^hum^* mice spontaneously developed large scrotal hernias (>200 mm^2^). Treatment of *Arom^hum^* mice with RU486 significantly reduced average hernia size or prevented hernia development entirely (Figure 2, B and C). WT littermates treated with Veh or RU486 did not exhibit changes in scrotal size. Histological analysis of LAM tissue after the 12-week treatment period showed significantly decreased fibrosis and increased average myofiber diameter in RU486-treated *Arom^hum^* mice compared with their Veh-treated counterparts (Figure 2, C–E).

We also sought to determine whether RU486 or UPA treatment could stall or reverse hernia growth after hernias have already developed. *Arom^hum^* mice were allowed to develop small-to-medium sized hernias (150–200 mm^2^) before being treated with RU486 or UPA for 12 weeks (Figure 2F). While the hernias of the Veh-treated *Arom^hum^* mice continued to grow during this time, the hernias of RU486-treated *Arom^hum^* mice remained roughly the same size, indicating that P4/PGR antagonists could stall hernia growth (Figure 2, G and H). Histological analysis of LAM tissue after the 12-week treatment period also showed decreased LAM fibrosis and increased LAM average myofiber diameter (Figure 2, H–J).

In these experiments, IHC analysis showed significantly increased stromal/fibroblast PGR expression in *Arom^hum^* LAM compared with WT LAM, with RU486 having no effect on PGR expression (Supplemental Figure 1; supplemental material available online with this article; https://doi.org/10.1172/jci.insight.193208DS1).

Similarly to RU486, treatment of *Arom^hum^* mice with UPA starting before hernia onset also resulted in decreased hernia size and LAM fibrosis, as well as increased LAM average myofiber diameter (Supplemental Figure 2, A–E). UPA treatment of *Arom^hum^* mice with established hernias also stalled further hernia growth and had similar findings on histology (Supplemental Figure 2, F–J). Taken together, the results from these experiments suggest that inhibition of PGR signaling in LAM fibroblasts prevents hernia development and stalls established hernia growth by attenuating LAM fibrosis and muscle atrophy.

### Inhibition of P4/PGR signaling prevents exogenous E2- plus P4-induced hernia development in WT mice.

**Figure 3:**
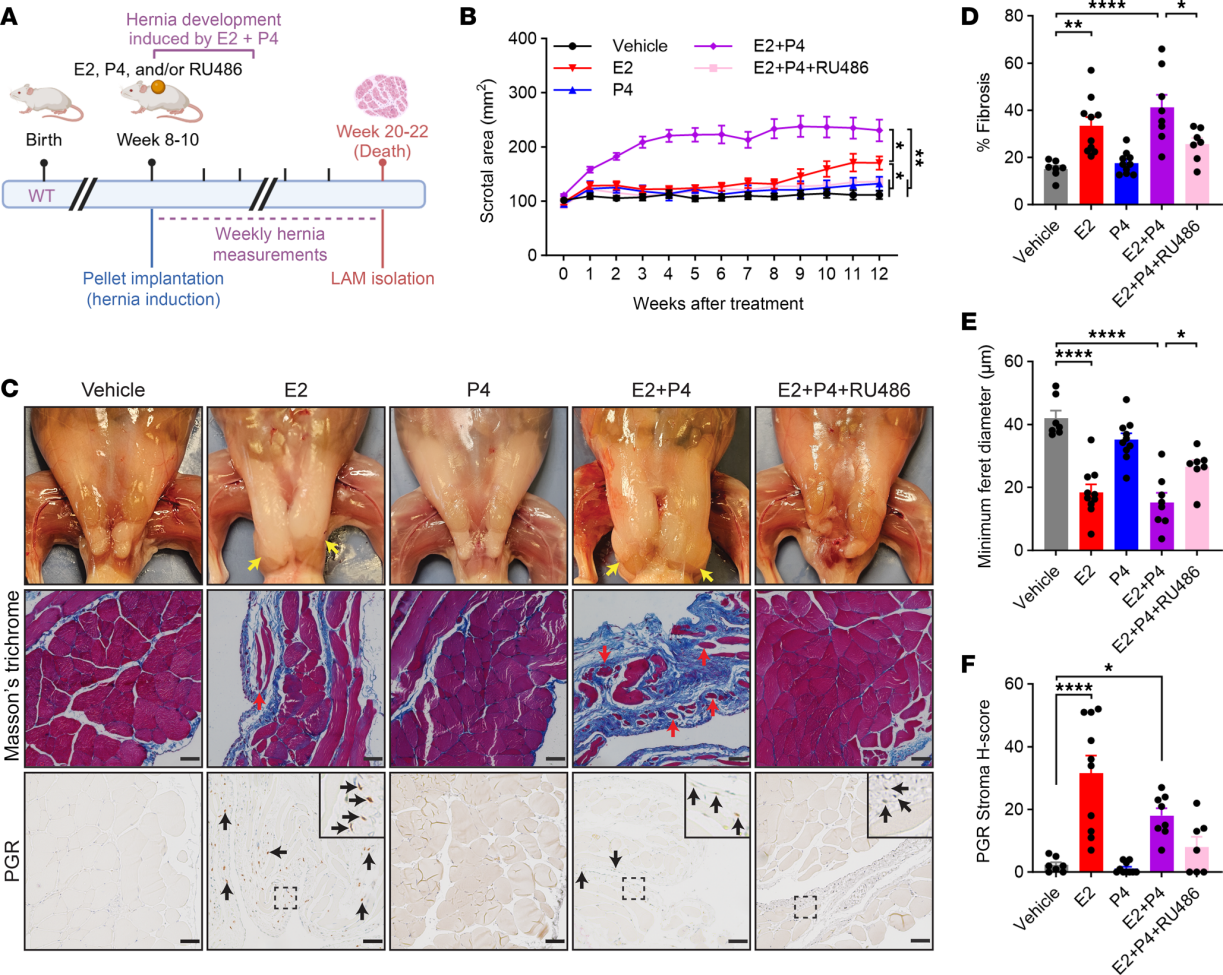
RU486 treatment prevents exogenous E2/P4-induced hernia development in WT mice. (**A**) Schematic for induced hernia development in WT mice by administration of E2, P4, and/or RU486. Created with BioRender.com. (**B**) Scrotal/hernia area measurements in WT mice treated with E2, P4, and/or RU486 as in **A** (*n* = 10–14/group, mean ± SEM, repeated-measures ANOVA). (**C**) Representative images of LAM morphology, Masson’s trichrome staining, and IHC staining for PGR in WT mice after 12 weeks of E2, P4, and/or RU486 treatment. Bilateral scrotal hernias (yellow arrows) and atrophying myofibers in herniated LAM tissue (red arrows) are highlighted for E2- and E2/P4-treated mice. (**D**–**F**) Quantification of (**D**) fibrotic area, (**E**) minimum Feret diameter, and (**F**) stromal PGR expression in WT mice after 12 weeks of E2, P4, and/or RU486 treatment (*n* = 7–10/group, mean ± SEM, 1-way ANOVA). Scale bars: 50 μm. *P < 0.05; **P < 0.01; ****P < 0.0001.

Although RU486 and UPA prevented hernia development in *Arom^hum^* mice, neither RU486 nor UPA are specific PGR antagonists; both drugs have mild affinity for other steroid hormone receptors such as glucocorticoid receptor (29, 30). Previous studies demonstrated that prolonged E2 administration could induce development of scrotal hernias in male mice (31). We sought to define the role of P4/PGR signaling in hernia development by administering E2 and/or P4 to WT mice for 12 weeks starting at 8–10 weeks of age (Figure 3A). WT mice given E2 alone began to develop small scrotal hernias (~150 mm^2^) after approximately 10 weeks of treatment, while WT mice given Veh or P4 alone did not develop hernias during the full 12-week treatment. Interestingly, WT mice given both E2 and P4 (EP) developed much larger scrotal hernias (>200 mm^2^) after only approximately 4 weeks of treatment. Concurrent administration of RU486 in these EP-treated mice prevented hernia development (Figure 3, B and C).

Histological examination of LAM tissue after 12 weeks showed that treatment with both E2 alone and EP resulted in significantly increased LAM fibrosis and decreased average myofiber diameter compared with treatment with Veh. These effects were attenuated by RU486 (Figure 3, C–E). Importantly, the LAM of E2- and EP-treated mice had increased PGR expression in the stromal compartment, indicating that E2 induces expression of PGR in LAM fibroblasts in vivo (Figure 3, C and F). Taken together, these results demonstrate the central role of P4/PGR signaling in LAM fibrosis, atrophy, and hernia development, while also confirming that the attenuating effects of RU486 treatment are mediated by P4/PGR.

### LAM tissue from EP-induced hernia mice contains a unique population of Pgr^+^ fibroblasts.

To determine the mechanisms through which P4/PGR signaling causes LAM fibrosis, muscle atrophy, and hernia development, we performed snRNA-seq of the LAM of WT mice after 10 weeks of treatment with Veh, EP, and E2 plus P4 plus RU486 (EPR) (Supplemental Figure 3A). LAM tissue was harvested from treated WT mice (*n* = 3–4 per group) and dissociated into single-nuclei suspensions, capturing the nuclei of both mononucleated and multinucleated cells.

**Figure 4:**
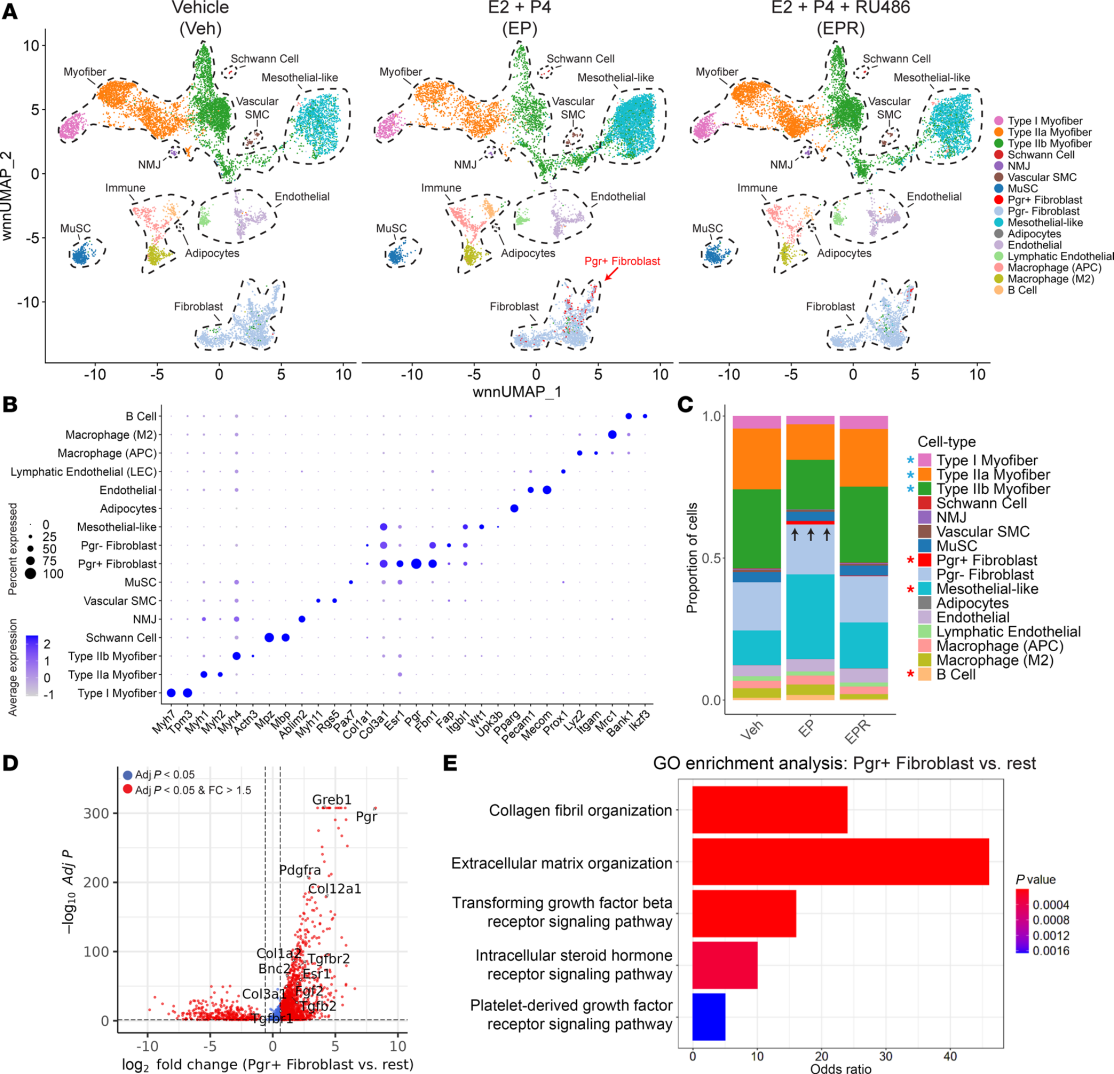
Single-nuclei RNA sequencing analysis reveals a unique population of *Pgr^+^* fibroblasts, increased mesothelial-like cells, and decreased myofibers in the LAM of mice with E2/P4-induced hernias. (**A**) Weighted nearest neighbor (WNN) UMAP plots showing cell types in the LAM of WT mice treated with vehicle (Veh), E2 + P4 (EP), and E2 + P4 + RU486 (EPR), normalized to 10,000 cells per sample to disclose proportional differences. *Pgr^+^* fibroblasts in EP LAM (red) are highlighted. NMJ, neuromuscular junction; SMC, smooth muscle cell; MuSC, muscle stem cell. (**B**) Dot plot showing the expression of known marker genes for individual cell types. Size of dots corresponds to frequency of expression within a cell group. Dot intensity corresponds to average expression level within the cell group. (**C**) Compositional makeup of the total cell population from Veh-, EP-, and EPR-treated LAM, represented as the proportion of total cells. Red and cyan asterisks indicate significantly increased or decreased cell types (comprising >1% of total EP LAM cells) in EP LAM compared with Veh and EPR LAM, respectively. Black arrows show increased *Pgr^+^* fibroblast population in EP LAM (fold change > 1.5, permutation test with FDR correction, *P* < 0.05 for EP vs. Veh or EPR). (**D**) Volcano plot showing upregulated and downregulated genes in *Pgr^+^* fibroblasts compared to other LAM cell types (fold change > 1.5, Wilcoxon’s rank-sum test, *P* < 0.05). (**E**) Gene Ontology (GO) processes enriched in *Pgr^+^* fibroblasts represented by odds ratio. *n* = 3–4/group.

After integration of nuclei data from all samples, cell type annotation was performed utilizing top differentially expressed genes (DEGs) as well as known marker genes (Figure 4, A and B). We identified all expected major cell types in LAM tissue, with the greatest proportion of nuclei from type I myofibers (*Myh7*, *Tpm3*), type IIa myofibers (*Myh1*, *Myh2*), type IIb myofibers (*Myh4*, *Actn3*), fibroblasts (*Fbn1*, *Fap*, *Itgbl1*), and mesothelial-like cells (*Wt1*, *Upk3b*). Other identified cell types included muscle stem cells, vascular smooth muscle cells, endothelial/lymphatic endothelial cells, immune cells (macrophages, B cells), Schwann cells, adipocytes, and neuromuscular junction–associated nuclei (Figure 4, A and B, and Supplemental Figure 3B).

By aggregating the expression values of all nuclei within each treatment group, we performed “pseudo-bulk” analysis to investigate differences in gene expression between Veh, EP, and EPR groups at the level of the entire LAM (32). We found 885 upregulated genes specific to EP LAM, whereas Veh and EPR LAM had considerable overlap in gene expression, with 107 common upregulated genes (Supplemental Figure 4, A and B). EP LAM had several upregulated genes involved in fibrosis (*Fgf1*, *Pdgfc*, *Pdgfd*, *Tgfbr2*, *Bnc2*, *Plod2*) (33–37) as well as several downregulated genes involved in normal skeletal muscle function (*Myh1*, *Myh4*, *Ttn*, *Dmd*) (38–40) (Supplemental Figure 4C). Pathway analysis of EP LAM revealed enrichment of genes involved in TGF-β receptor (TGFBR), platelet-derived growth factor receptor (PDGFR), epidermal growth factor receptor (EGFR), and interleukin-6–mediated (IL-6–mediated) signaling, suggesting that at the overall tissue level, EP LAM is more proinflammatory and profibrotic compared with Veh and EPR LAM (Supplemental Figure 4D).

At the individual cell/nucleus level, the LAM of WT mice in the EP group displayed noticeable differences in cell type distribution compared with the Veh and EPR groups (Figure 4A). Cell type proportions for Veh, EP, and EPR LAM were analyzed as the proportion of the total number of cells per sample (Figure 4C). Of the cell types in each treatment group that made up a significant number (>1%) of the total cell population, EP LAM contained significantly higher proportions of mesothelial-like cells and B cells compared with both Veh and EPR LAM, as well as significantly lower proportions of type I, type IIa, and type IIb myofibers (Figure 4C and Supplemental Figure 5, A and B). Cell type proportions in Veh and EPR LAM looked similar overall, except for a higher proportion of M2 macrophages in Veh LAM among the cells with significant (>1%) numbers (Supplemental Figure 5C).

Importantly, we identified a unique population of fibroblasts that was only present in significant numbers (>1%) in EP LAM. These fibroblasts were characterized by high expression of *Pgr* and thus termed “*Pgr^+^* fibroblasts” (Figure 4C). Other fibroblasts that did not express *Pgr* were termed *Pgr^–^* fibroblasts. DEG analysis of *Pgr^+^* fibroblasts versus other cell types found in the LAM revealed several upregulated fibrosis-associated genes, including multiple collagens (*Col1a2*, *Col3a1*, *Col12a1*), ligands/receptors for profibrotic signaling pathways (*Tgfb2*, *Tgfbr1*, *Tgfbr*2, *Pdgfra*) (34, 35), and profibrotic transcription factors (*Bnc2*) (36) (Figure 4D). *Pgr^+^* fibroblasts also exhibited the highest expression of *Esr1* and *Pgr* of any cell type (Figure 4, B and D). Pathway analysis of *Pgr^+^* fibroblasts revealed enrichment of genes associated with TGF-β, PDGF, and intracellular steroid hormone signaling pathways, as well as enrichment of genes associated with collagen fibril organization and ECM organization (Figure 4E). Taken together, these results suggest that the extensive LAM fibrosis in EP mice is driven, at least in part, by a unique population of fibroblasts that are highly activated and responsive to both E2 and P4.

### Pgr^+^ fibroblasts may communicate with Pgr^–^ fibroblasts and mesothelial-like cells to promote profibrotic behavior.

**Figure 5:**
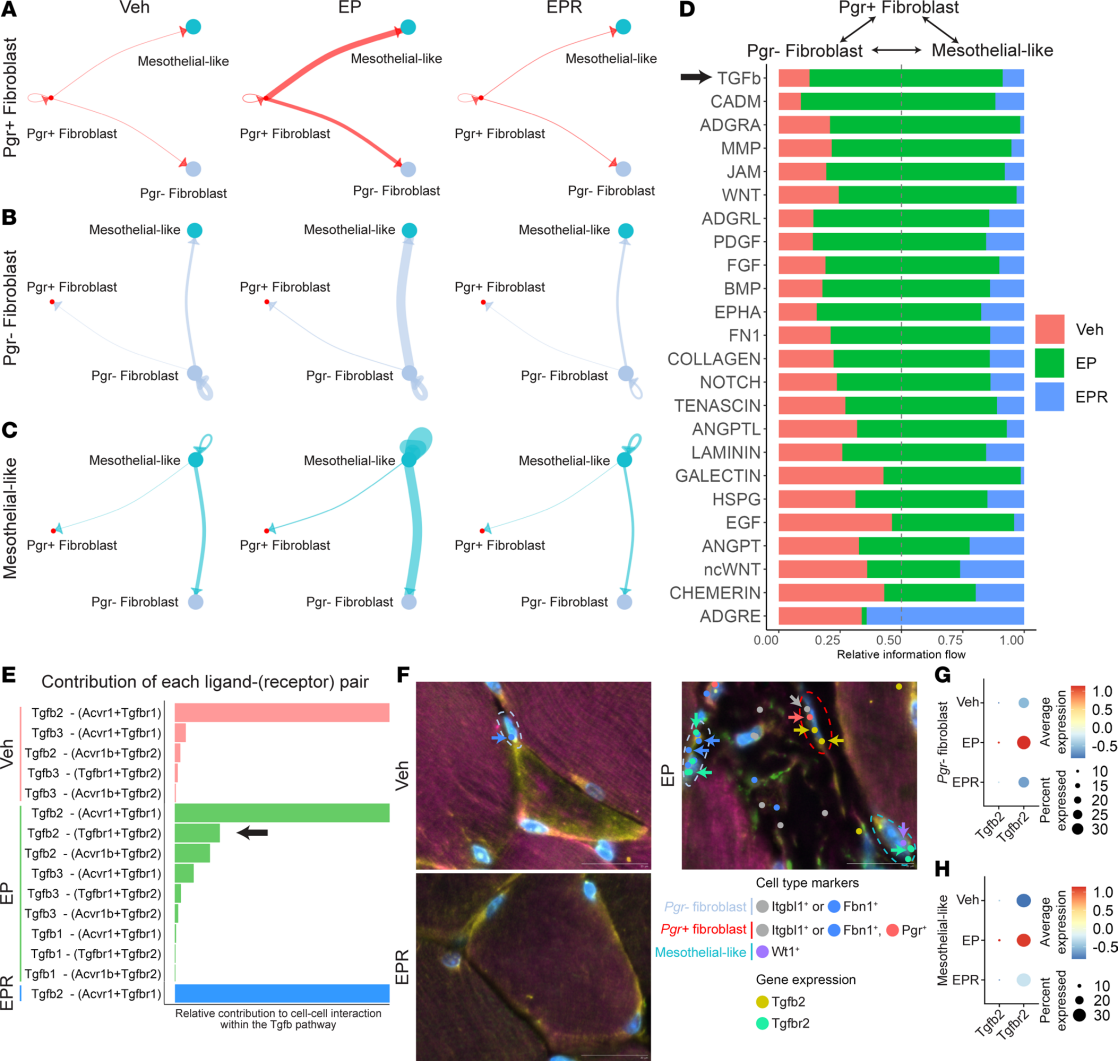
*Pgr^+^* fibroblasts may communicate with *Pgr^–^* fibroblasts and mesothelial-like cells to promote profibrotic behavior. (**A**–**C**) Visualization of cell-cell communication between *Pgr^+^* fibroblasts, *Pgr^–^* fibroblasts, and mesothelial-like cells using CellChat, comparing communication networks between Vehicle (Veh), E2 + P4 (EP), and E2 + P4 + RU486 (EPR) LAM. Circle plots with (**A**) *Pgr^+^* fibroblasts, (**B**) *Pgr^–^* fibroblasts, and (**C**) mesothelial-like cells as the central outgoing nodes. Interactions between pairs of cell types are depicted by a line connecting the 2 cell types, with line thickness indicating the strength of the interaction. (**D**) Signaling pathways enriched within the *Pgr^+^* fibroblast, *Pgr^–^* fibroblast, and mesothelial-like communication network of Veh, EP, and EPR LAM. Black arrow emphasizes the TGF-β pathway. (**E**) Relative contribution of TGF-β pathway ligand-receptor pairs in Veh (red), EP (green), and EPR (blue) LAM. Upregulated Tgfb2-(Tgfbr1+Tgfbr2) interaction in EP LAM is highlighted (black arrow). (**F**) Xenium-based spatial transcriptomics showing localization of *Tgfb2*-expressing *Pgr^+^* fibroblasts (*Fbn1^+^* or *Itgbl1^+^*, *Pgr^+^*; red dashed circle) with *Tgfbr2*-expressing *Pgr^–^* fibroblasts (*Fbn1^+^* or *Itgbl1^+^*, *Pgr^–^*; light gray dashed circle), and mesothelial-like cells (*Wt1^+^*; cyan dashed circle) in EP LAM, but not in the Veh or EPR LAM controls. The software uses DAPI to determine the extent of the nucleus (light blue staining). Colored dots, each representing an individual mRNA transcript of a gene, are emphasized with similarly colored arrows. (**G** and **H**) Comparison of *Tgfb2* and *Tgfbr2* expression in Veh, EP, and EPR LAM for (**G**) *Pgr^–^* fibroblasts and (**H**) mesothelial-like cells on Xenium-based spatial transcriptomics. Scale bars: 20 μm. *n* = 3–4/group.

To determine how *Pgr^+^* fibroblasts and other cell types interact, we surveyed intercellular communication networks using CellChat (41). The probability of communication between 2 cell groups was visualized with circle plots, with thicker lines between cell types representing a higher degree of inferred communication. Outgoing ligands/communication from *Pgr^+^* fibroblasts to other cell types was assessed by designating *Pgr^+^* fibroblasts as the central node in our dataset. In EP LAM, *Pgr^+^* fibroblasts had the highest probability of communication with *Pgr^–^* fibroblasts and mesothelial-like cells, followed by communication with myofibers (Figure 5A and Supplemental Figure 6A). The probability of this communication was much lower in Veh and EPR LAM (Figure 5A). Similar surveys of intercellular communication networks were performed with *Pgr^–^* fibroblasts and mesothelial-like cells as the central nodes. These cell types exhibited the highest communication probability with themselves and each other, especially in EP LAM (Figure 5, B and C, and Supplemental Figure 6, B and C). However, communication initiating from *Pgr^–^* fibroblasts and mesothelial-like cells to *Pgr^+^* fibroblasts was limited, suggesting that *Pgr^+^* fibroblasts are upstream regulators of *Pgr^–^* fibroblasts and mesothelial-like cells.

Information flow for significant signaling pathways between these 3 cell types was also assessed (Figure 5D). Some pathways (e.g., ANGPT, ncWNT, chemerin) maintained similar information flow in Veh, EP, and EPR LAM, suggesting that these signaling pathways do not change significantly between healthy and fibrotic LAM. Other pathways had significantly higher information flow in EP LAM compared with Veh and EPR LAM, including several fibrosis-associated signaling pathways (TGF-β, PDGF, FGF) and ECM-associated signaling pathways (MMP, collagen).

The TGF-β signaling pathway, a well-established driver of fibroblast activation and fibrosis (42), was highly predominant in EP LAM compared with other signaling pathways (Figure 5D). Several TGF-β–related ligands/receptors (*Tgfb2*, *Tgfbr1*, *Tgfbr*2) were also upregulated in *Pgr^+^* fibroblasts (Figure 4D). For these reasons, we utilized CellChat to determine the relative contribution of each TGF-β pathway ligand-receptor (L-R) pair utilized in communication between *Pgr^+^* fibroblasts, *Pgr^–^* fibroblasts, and mesothelial-like cells (Figure 5E). In this pathway, a TGF-β ligand binds to a complex consisting of 2 receptor isoforms [L-(R+R)] (42). The Tgfb2-(Acvr1+Tgfbr1) interaction exhibited a consistently high contribution across Veh, EP, and EPR LAM, suggesting a similar basal level of signaling activity involving this L-R pair (Figure 5E). However, the Tgfb2-(Tgfbr1+Tgfbr2) interaction demonstrated a significantly higher relative contribution in EP LAM compared with Veh and EPR LAM (Figure 5E). This higher contribution was further supported by DEG analysis, which showed upregulation in EP LAM of *Tgfbr2* in *Pgr^+^* fibroblasts, *Pgr^–^* fibroblasts, and mesothelial-like cells, as well as *Tgfb2* in *Pgr^+^* fibroblasts and *Pgr^–^* fibroblasts (Figure 4D and Supplemental Figure 7, A and B). Additionally, inferred motif activity of the TGF-β downstream transcription factors Smad2 and Smad3 was increased in EP LAM compared with Veh and EPR LAM in *Pgr^–^* fibroblasts and mesothelial-like cells (Supplemental Figure 7, C and D) (42). *Pgr^+^* fibroblasts in EP LAM also exhibited high inferred motif activity of Smad2/Smad3, consistent with the other mentioned cell types in this communication network (Supplemental Figure 7E). These findings suggest that the Tgfb2-(Tgfbr1+Tgfbr2) L-R pair may play a larger role in communication between these cell types in EP LAM.

Additionally, we performed Xenium-based spatial transcriptomics to further analyze the expression of *Tgfb2* and *Tgfbr2* in these cell types (Supplemental Figure 8, A and B). In our spatial transcriptomics dataset, *Pgr^+^* fibroblasts were only found in EP LAM, and *Pgr^–^* fibroblasts and mesothelial-like cells were increased in EP LAM compared with Veh and EPR LAM (Supplemental Figure 8, C and D). Spatially resolved expression of *Tgfb2* and *Tgfbr2* was significantly increased in EP LAM compared with Veh and EPR LAM, with particularly high expression in the inferior LAM portion where herniation occurs (Supplemental Figure 8, E and F). Spatial transcriptomics also showed *Pgr^+^* fibroblasts (*Fbn1^+^* or *Itgbl1^+^*, *Pgr^+^*) expressing *Tgfb2* in close proximity with *Pgr^–^* fibroblasts (*Fbn1^+^* or *Itgbl1^+^*, *Pgr^–^*) and mesothelial-like cells (*Wt1^+^*) expressing *Tgfbr2* in EP LAM, providing further evidence for the importance of TGF-β pathway signaling among these 3 cell types (Figure 5F). This colocalization was not present in Veh or EPR LAM (Figure 5F). The expression levels of *Tgfb2* and *Tgfbr2* in our spatial transcriptomics dataset were also higher in *Pgr^–^* fibroblasts and mesothelial-like cells in EP LAM compared with Veh and EPR LAM (Figure 5, G and H). These findings provide further evidence for the potential role of TGF-β2/TGFBR2 signaling in mediating communication between these cell types.

We further sought to characterize the difference between *Pgr^–^* fibroblasts and mesothelial-like cells in EP LAM compared to Veh and EPR LAM. DEG analysis revealed several upregulated profibrotic genes in EP LAM for these cell types, including *Zbtb16*, *Sfrp4*, and *Plod2* (37, 43, 44) in *Pgr*^–^fibroblasts and *Thbs1*, *Pim1*, and *Foxo1* (45–47) in mesothelial-like cells (Supplemental Figure 9, A and B). *Pgr^–^* fibroblasts and mesothelial-like cells in EP LAM also had increased expression of *Pdgfc* and *Pdgfd*, encoding 2 growth factors involved in fibroblast activation (34, 48, 49). Pathway analysis revealed an enrichment of genes associated with response to TGF-β, the PDGFR signaling pathway, cell migration, and epithelial-mesenchymal transition for both *Pgr^–^* fibroblasts and mesothelial-like cells in EP LAM (Supplemental Figure 9C). *Pgr^–^* fibroblasts were also enriched in genes associated with ECM organization and collagen fibril organization (Supplemental Figure 9C). Collectively, these results suggest that cell-cell communication among *Pgr^+^* fibroblasts, *Pgr^–^* fibroblasts, and mesothelial-like cells via the TGF-β pathway contributes to activation and profibrotic gene expression of these cells in EP LAM.

### Pgr^+^ fibroblasts, Pgr^–^ fibroblasts, and mesothelial-like cells may contribute to atrophy of LAM myofibers in EP-induced hernia mice.

**Figure 6:**
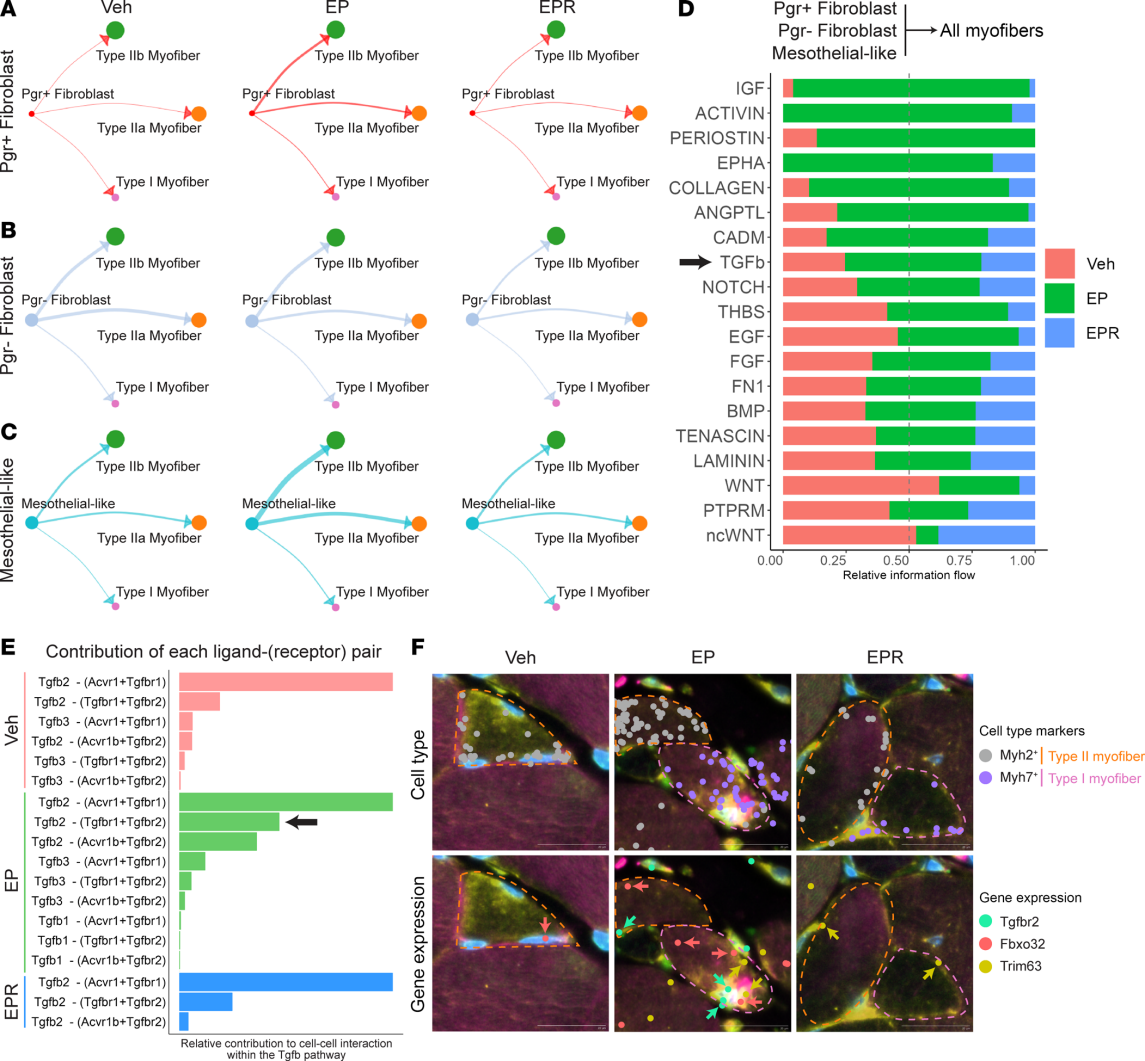
*Pgr^+^* fibroblasts, *Pgr^–^* fibroblasts, and mesothelial-like cells may contribute to atrophy of myofibers in EP LAM. (**A**–**C**) Visualization of cell-cell communication using CellChat, comparing communication networks between Vehicle (Veh), E2 + P4 (EP), and E2 + P4 + RU486 (EPR) LAM. Circle plots visualizing networks with outgoing communication from (**A**) *Pgr^+^* fibroblasts, (**B**) *Pgr^–^* fibroblasts, and (**C**) mesothelial-like cells to type I/IIa/IIb myofibers. Interactions between pairs of cell types are depicted by a line connecting the 2 cell types, with line thickness indicating the strength of the interaction. (**D**) Signaling pathways enriched in Veh, EP, and EPR LAM within the communication network between non-myofibers (*Pgr^+^* fibroblasts, *Pgr^–^* fibroblasts, mesothelial-like cells) and type I/IIa/IIb myofibers. Black arrow emphasizes the TGF-β pathway. (**E**) Relative contribution of TGF-β pathway ligand-receptor pairs in Veh (red), EP (green), and EPR (blue) LAM. Upregulated Tgfb2-(Tgfbr1+Tgfbr2) interaction in EP LAM is highlighted (black arrow). (**F**) Xenium-based spatial transcriptomics of Veh, EP, and EPR LAM showing expression of *Tgfbr2* and muscle atrophy markers (*Fbxo32*, *Trim63*) in type I (*Myh7^+^*; pink dashed circle) and type II myofibers (*Myh2^+^*, orange dashed circle). Upper row images show expression of cell-type marker genes, while lower row images show expression of *Tgfbr2* and muscle atrophy marker genes (*Fbxo32*, *Trim63*). The software uses DAPI to determine the extent of the nucleus (light blue staining). Colored dots, each representing an individual mRNA transcript of a gene, are emphasized with similarly colored arrows. Scale bars: 20 μm. *n* = 3–4/group.

Because our CellChat analysis showed that *Pgr^+^* fibroblasts, *Pgr^–^* fibroblasts, and mesothelial-like cells also communicate extensively with myofibers (Supplemental Figure 6), we sought to determine whether this communication affects myofiber behavior. Cell-cell communication probabilities were analyzed by pairing ligands from *Pgr^+^* fibroblasts, *Pgr^–^* fibroblasts, and mesothelial-like cells with receptors from type I, type IIa, and type IIb myofibers (Figure 6, A–C). Within this network, the TGF-β signaling pathway was also one of several pathways that showed higher information flow in EP LAM compared with Veh and EPR LAM (Figure 6D).

Because TGF-β signaling has also been shown to contribute to myofiber atrophy, we further characterized this pathway within the network (42). Inspection of the relative contribution of each L-R pair within this pathway revealed that all 3 treatment groups once again had high basal levels of signaling activity involving the Tgfb2-(Acvr1+Tgfbr1) interaction, while EP LAM had increased signaling activity involving the Tgfb2-(Tgfbr1+Tgfbr2) interaction (Figure 6E). Myofibers from EP LAM had significantly higher expression of *Tgfbr2* and higher inferred Smad2/Smad3 motif activity compared with myofibers in Veh and EPR LAM, suggesting that the Tgfb2-(Tgfbr1+Tgfbr2) L-R pair may play a more prominent role within EP LAM myofibers (Supplemental Figure 10, A and B) (42). Spatial transcriptomics showed myofibers expressing *Tgfbr2* in EP LAM, but not in Veh or EPR LAM (Figure 6F). Notably, these *Tgfbr2^+^* myofibers also expressed *Fbxo32* and *Trim63*, 2 well-known muscle atrophy–associated genes (Figure 6F) (50–52).

We further characterized gene expression differences of type I, type IIa, and type IIb myofibers in EP LAM compared to Veh and EPR LAM. DEG analysis showed that in EP LAM, myofibers had high expression levels of atrophy-associated genes, including *Fbxo32*, *Trim63*, *Pdk4*, *Foxo1*, and *Foxo3* (Supplemental Figure 11, A–C) (50–53). Pathway analysis of all 3 myofiber types also showed enrichment of genes involved in protein ubiquitination and catabolism (Supplemental Figure 11D). Myofibers in EP LAM were also enriched in genes associated with response to TGF-β signaling (Supplemental Figure 11D). Taken together, these findings, along with the decreased myofiber cross-sectional area (CSA) (Figure 3, C and E) and proportion (Figure 4C and Supplemental Figure 5, A and B) in EP LAM compared with Veh and EPR LAM, suggest that EP LAM myofibers are undergoing atrophy and that *Pgr^+^* fibroblasts, *Pgr^–^* fibroblasts, and mesothelial-like cells may contribute to this process through TGF-β pathway signaling.

### Increased expression of TGF-β pathway–related genes and myofiber atrophy–associated genes in human hernia tissue.

**Figure 7:**
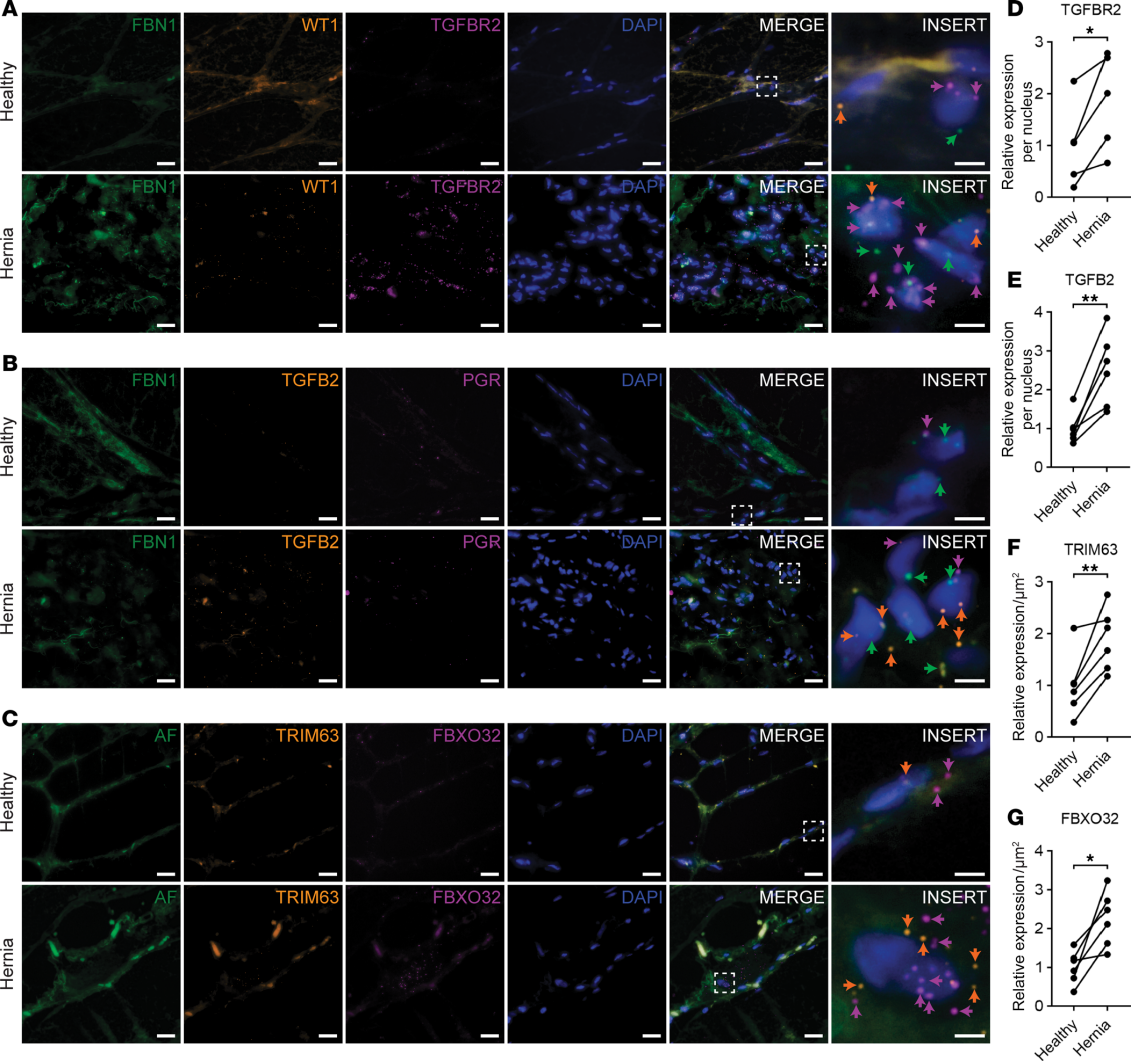
Increased expression of TGF-β pathway–related genes and myofiber atrophy–associated genes in human hernia tissue. (**A**–**C**) Representative RNAscope images from herniated and adjacent healthy LAM tissue from male patients showing (**A**) expression of *TGFBR2* (magenta arrow) in fibroblasts (*FBN1^+^*, green arrow) and mesothelial-like cells (*WT1^+^*, orange arrow), (**B**) expression of *TGFB2* (orange arrow) in *PGR^+^* (*FBN1^+^*, green arrow; *PGR^+^*, magenta arrow) and *PGR^–^* (*FBN1^+^*, green arrow; *PGR^–^*) fibroblasts, and (**C**) expression of *TRIM63* (orange arrow) and *FBXO32* (magenta arrow) in myofibers (green outline in autofluorescent (AF) channel). (**D**–**G**) Quantification of expression levels of (**D**) *TGFBR2*, (**E**) *TGFB2*, (**F**) *TRIM63*, and (**G**) *FBXO32* in healthy and herniated LAM tissue from male patients (*n* = 5–6/group, paired *t* test). Scale bars: 25 μm and 5 μm (INSERT). *P < 0.05, **P < 0.01.

To determine whether the changes in gene expression and cell-cell communication found in mice also occur in humans, we employed multiplex RNA in situ hybridization on herniated/fibrotic LAM tissue and matched adjacent healthy LAM tissue from male patients. Utilizing this approach, we identified *PGR^+^* fibroblasts (*FBN1^+^*, *PGR^+^*), *PGR^–^* fibroblasts (*FBN1^+^*, *PGR^–^*), and mesothelial-like cells (*WT1^+^*) in herniated LAM tissue (Figure 7, A and B). Furthermore, we identified *PGR^–^* fibroblasts and mesothelial-like cells that expressed *TGFBR2* and *PGR^+^/PGR^–^* fibroblasts that expressed *TGFB2* (Figure 7, A and B). Expression levels of both *TGFBR2* and *TGFB2* were significantly increased in herniated LAM tissue compared with matched healthy LAM tissue (Figure 7, D and E), suggesting that increased TGF-β signaling in LAM tissue may contribute to hernia development in humans.

We also analyzed the expression of *FBXO32* and *TRIM63* in the myofibers of both healthy and herniated LAM tissue (Figure 7C). Expression levels of both *FBXO32* and *TRIM63* were significantly increased in herniated LAM tissue compared with matched healthy LAM tissue (Figure 7, F and G). Taken together, the increased expression of *TGFB2*, *TGFBR2*, *FBXO32*, and *TRIM63* provides additional evidence for the role of TGF-β signaling as a potential downstream mechanism for EP-induced abdominal fibrosis and muscle atrophy.

## Discussion

Most research on P4/PGR signaling focuses on its role in women rather than men, despite similar serum P4 levels in men and women outside the luteal phase (27). Here, we revealed an important pathologic role of P4/PGR signaling in men by showing that exogenous P4 administration together with E2 can lead to hernia formation in male WT mice, with EP treatment resulting in increased hernia size and more rapid hernia development compared with E2 treatment alone. The similarities in LAM morphology between the E2- and EP-treated mice can be explained by the presence of progestogenic action in E2-treated mice from endogenously produced P4 (Figure 1B) rather than exogenously administered P4. The use of this model in tandem with the established *Arom^hum^* hernia model paints a more complete picture of the complex relationship between sex steroid signaling, skeletal muscle fibrosis/atrophy, and hernia pathogenesis.

Inguinal hernia development has been associated with atrophy and fibrosis of LAM tissue in male hernia patients (11). Furthermore, elderly men, a demographic disproportionately affected by inguinal hernias, are known to have relatively higher serum E2 levels compared with younger men (54–56). Both of our mouse models mimic this increase in E2, either through endogenous production in *Arom^hum^* mice or exogenous E2 administration in WT mice, thus increasing the clinical translatability of our findings (12, 19, 20). The herniated LAM tissues from these mouse models, which exhibit extensive muscle atrophy and fibrosis, are also histologically similar to the resected muscle tissue of male patients undergoing hernia repair surgery (Figure 1E and Figure 2C).

While the *Arom^hum^* hernia model has been used previously to establish the role of E2/ESR1 signaling in LAM fibroblast activation, this study furthers our understanding of hernia pathogenesis by implicating P4/PGR signaling as an important downstream regulator of E2/ESR1-induced LAM fibrosis (12, 19, 20). The relationship between E2/ESR1 signaling and PGR expression is well established in the breast, ovary, and uterine endometrium (21–23, 57). We are the first to our knowledge to demonstrate that this relationship holds true in skeletal muscle, particularly in LAM fibroblasts. We also showed that E2 and R5020, a P4 analogue, can synergistically increase LAM fibroblast proliferation (Figure 1D), consistent with previous studies examining E2 and R5020 action on cultured fibroblasts in uterine leiomyoma (26). Pharmacologic antagonism of P4/PGR signaling via RU486 and UPA decreased LAM fibroblast proliferation in vitro (Figure 1D) and decreased LAM fibrosis, muscle atrophy, and hernia formation in vivo (Figure 2 and Supplemental Figure 2). Furthermore, we demonstrated that RU486 exerts its effects specifically through antagonism of PGR, as RU486 prevented hernia formation induced by EP treatment in male WT mice (Figure 3, B and C). Currently, RU486 and UPA are primarily used for pregnancy termination or emergency contraception in women (58, 59). Our findings, along with the positive correlation between ESR1, PGR, and Ki-67 expression in human stromal hernia tissue (Figure 1, E and F), highlight both a role for P4/PGR signaling and a potential use for PGR antagonists in men.

Fibroblasts play a key role in LAM fibrosis through their ability to synthesize and modulate components of the ECM. Under pathological states, fibroblasts alter their transcriptional signature to increase synthesis and secretion of ECM proteins, cytokines, and growth factors, resulting in aberrant buildup of fibrotic stroma (60, 61). Utilizing snRNA-seq of LAM tissue, we found that exogenous EP treatment of WT mice leads to similar changes in the transcriptional signature of LAM fibroblasts that contribute to LAM fibrosis, including upregulation of several profibrotic genes (e.g., *Zbtb16*, *Sfrp4*, and *Plod2*) (37, 43). Of note, expression of the transcription factor *Zbtb16* in fibroblasts has been shown to contribute to fibrosis of the heart, lung, and liver in mouse/rat models (44, 62, 63). ZBTB16 is also expressed in human fibroblasts, with dysregulation of ZBTB16 expression linked to aging in otherwise healthy human individuals (64, 65). *Zbtb16* has not been studied in skeletal muscle, but further investigation could offer insight into its role as a mediator of LAM fibrosis and as a possible age-related risk factor for hernia development.

While fibroblasts are traditionally seen as the primary drivers of fibrosis, several other cell types, including immune cells, endothelial cells, and mesothelial cells, may also contribute to this process (66–68). In particular, mesothelial cells are an interesting yet understudied contributor to fibrosis (69). Mesothelial cells and fibroblasts in the lung communicate via TGF-β and Wnt signaling to promote fibrosis in models of idiopathic pulmonary fibrosis (70). Mesothelial cells may also take a more direct role in lung, liver, and peritoneal fibrosis by transforming into a fibroblast-like cell in a process called mesothelial-mesenchymal transition (71–74). Our results suggest that mesothelial-like cells may play a similar role in LAM fibrosis by communicating with fibroblasts and myofibers via TGF-β and/or PDGF signaling, although further studies are needed. Lastly, *WT1*, a gene highly expressed in mesothelial-like cells in our dataset and also present in hernia tissue from male patients, has been linked to inguinal hernia susceptibility in humans, further warranting additional investigation into the role of mesothelial-like cells in LAM fibrosis (75).

Cell-cell communication plays a central role in promoting tissue fibrosis. The TGF-β signaling pathway in particular is a well-established driver of fibrosis of multiple organ systems, including the skin, liver, lung, and skeletal muscle (42, 76–79). Our analyses in both mouse EP LAM and human hernia tissue provide evidence for the involvement of TGF-β signaling in LAM fibrosis and hernia development, particularly through TGF-β2. Although TGF-β1 is the more well-established profibrotic ligand in the TGF-β signaling pathway, TGF-β2 binds and signals through the same receptors and has also been implicated in skin and lung fibrosis (80–83). *Pgr^+^* fibroblasts likely play a central role in this process of hernia development, as they are upregulated in EP LAM and have increased expression of *Tgfb2*, *Tgfbr1*, and *Tgfbr2*. Additionally, the presence of fibroblasts that coexpress *PGR* and *TGFB2* in human hernia tissue suggests that *Pgr^+^* fibroblast-driven TGF-β2 secretion could play a role in human LAM fibrosis. PGR has been shown to modulate the TGF-β pathway; for example, P4/PGR signaling increases TGF-β1 secretion in uterine epithelial cells (84). Antagonism of P4/PGR with UPA was also shown to decrease TGF-β signaling in uterine leiomyoma by downregulating TGFBR1 and TGFBR2 (85). Our findings expand on this relationship between PGR and TGF-β to include its action in skeletal muscle fibroblasts.

Although single-cell analyses of LAM have been performed previously in mouse models of hernia, our study is the first to our knowledge to analyze gene expression at the single-nucleus level, allowing us to capture and examine the behavior of myofibers/myonuclei (19, 86). In skeletal muscle, aberrant expansion of the ECM stroma is often accompanied by atrophy of myofibers, such as in muscular dystrophies, age-related sarcopenia, or severe injury (87). One of the hallmarks of muscle atrophy involves the expression of a series of genes involved in protein catabolism (50, 51). Of note, FBXO32 and TRIM63, 2 different ubiquitin ligases, are well-known atrophy-associated genes upregulated in the myofibers of EP LAM (50–52). In skeletal muscle, expression of FBXO32 and TRIM63 has been shown to be induced by both TGF-β1 and myostatin, another ligand in the TGF-β family (88–90). Our data suggest that TGF-β2 may play a similar role in inducing LAM myofiber atrophy and provide a potential mechanism for how fibroblasts and mesothelial cells can facilitate this process. Further investigation, such as inhibition of *Tgfb2/Tgfbr2* signaling in LAM fibroblasts and myofibers either in vitro or in vivo, is required to definitively show the role of TGF-β and other signaling pathways in cell-cell communication between these populations.

Our present study focuses on fibroblasts, mesothelial-like cells, and myofibers, as these were the cell types that made up the highest proportion of our captured cell population in single-nuclei sequencing. Other captured cell types, including endothelial cells, macrophages, and muscle stem cells, likely also play a role in LAM fibrosis and muscle atrophy. However, these populations were captured in fewer numbers. This shortcoming, combined with the inherent inability of single-nuclei sequencing to capture cytoplasmic mRNA, limited our ability to capture the heterogeneity within these cell types and view differences in their gene expression between treatment groups (91–93). Future experiments investigating the role of these cell types in LAM fibrosis may require enrichment for the particular cell population followed by single-cell/-nuclei sequencing. Another limitation in our sequencing experiments was the low quality of our ATAC data, which had consistently low TSS enrichment scores and high nucleosome signal, regardless of treatment group. As a result, we were unable to utilize the ATAC data for more than cell clustering and transcription factor activity inference. Future experiments seeking to analyze chromatin accessibility may consider using an alternative ATAC-seq modality instead.

Collectively, these findings illustrate the complex processes that contribute to LAM fibrosis and establish PGR signaling as a driver of hernia development as well as a potential target for pharmacologic management of inguinal hernia.

## Methods

Further information can be found in Supplemental Methods.

### Sex as a biological variable

The hernia phenotype seen in *Arom^hum^* mice and EP-treated mice occurs only in males. Incidence of inguinal hernias in humans is also 9–10 times greater in males than females. For these reasons, experiments were performed only in male mice and samples taken only from male patients.

### Mouse experiments

#### Mouse models.

Both WT and *Arom^hum^* mice used in experiments were FVB/N strain. To create *Arom^hum^* mice, bacterial artificial chromosome (BAC) DNA with the aromatase gene coding region, promoter regions, and 3′-polyadenylation site was created and injected into FVB/N-fertilized oocytes (Genetically Engineered Mouse Core, Baylor College of Medicine) (12, 19). Mice were maintained with a 14-hour light/10-hour dark cycle and given standard chow (Envigo, Teklad LM-485, 7912 for nonbreeders; S-2335, 7904 for breeders). Genotyping was performed according to previous studies (12). Specific genotyping PCR primers include *Arom^hum^* F (AGTATCCCGGTGGAGTGATCT) and *Arom^hum^* R (AAGCTGGCTGAAAGTCTAGGG). Scrotal areas were measured using a digital caliper (CSA [mm^2^] = length [mm] × width [mm]) in the morning, 2–3 times per week by a single experimenter to reduce variability. Mice were euthanized with ketamine-xylazine cocktail (100 mg/kg, 10 mg/kg) followed by cervical dislocation. Lower abdominal muscle was harvested as described previously (12).

#### Drug pellets.

Estradiol (E2, 0.3 mg/pellet, 90-day release, E-121) and progesterone (P4, 75 mg/pellet, 90-day release, P-131) slow-release pellets and their placebos (C-111) were purchased from Innovative Research of America. Mifepristone (RU486; Millipore Sigma, M8046) and UPA (AdooQ BioScience, A21041) were formulated into slow-release pellets (37.5 mg/pellet for mifepristone and 22.5 mg/pellet for UPA, 90-day release, X-999) and placebos (C-111) by Innovative Research of America.

#### Subcutaneous pellet implantation.

Mice were anesthetized and slow-release drug pellets were subcutaneously inserted into the side of the neck. For prevention studies, pellets were inserted in mice at 3–4 weeks of age. For hernia growth stalling studies, pellets were inserted once hernias were formed (150–200 mm^2^, ~6–10 weeks of age). For WT hernia induction studies, pellets were inserted in mice at 8–10 weeks of age.

### Human samples

Two biopsy specimens were obtained (1 × 0.5 × 0.5 cm^3^) per patient from a total of 43 patients, one from the hernia site and another from adjacent healthy muscle. None of the patients were receiving hormone therapy when tissues were collected. Average age was 50.76 years (SD ± 14.35 years, range 28–80 years). Average weight and height at the time of surgery were 77.68 kg (SD ± 14.78 kg) and 67.04 inches (SD ± 3.40 inches), respectively. 53.5% of patients underwent right inguinal hernia surgery and 46.5% underwent left inguinal hernia surgery.

### Histology and IHC

Histological and IHC experiments were conducted at the Northwestern University Mouse Histology and Phenotyping Laboratory core.

#### H&E and Masson’s trichrome staining.

LAM tissues were dissected and fixed in 4% paraformaldehyde in PBS for 24 hours at 4°C. Tissues were subsequently embedded in paraffin and sectioned at 4 μm thickness. Sections were stained with H&E as well as Masson’s trichrome (Weigert’s Hematoxylin, Biebrich Scarlet-Acid Fuchsin solution, and Aniline Blue) using a staining kit (American Master Tech, KTTRBPT). Images were obtained using an EVOS M5000 microscope (Thermo Fisher Scientific) at ×20 magnification. Tissue fibrotic area and myofiber minimum Feret diameter were quantified using ImageJ (NIH) color deconvolution and measure/analyze particles functions.

#### IHC.

LAM tissues were fixed, paraffin-embedded, and sectioned at 4 μm thickness. After deparaffinization and citrate antigen retrieval (Thermo Fisher Scientific, 50843064), sections were incubated with primary antibodies against ESR1 (1:100; Biocare SP1, OAA-301-T60 for human), Ki-67 (1:400; Abcam, ab15580 for human), or PGR (Dako, M3569 for human [1:400] or Abcam, ab63605 for mouse [1:500]) overnight at 4°C. Sections were then washed and incubated with secondary HRP-conjugated antibodies (Vector Laboratories). DAB (Dako, GV825) was used for chromogenic staining. Images were obtained using an EVOS M5000 microscope (Thermo Fisher Scientific) at ×20 magnification. IHC scoring was performed by an independent pathologist with blinding to sample type and treatment. Cells within samples were divided into skeletal muscle cells/fibers and stromal cells and scored separately. H-score was derived from a weighted categorization of weak, moderate, and strong staining.

### Single-nuclei multiomics sequencing

#### Single nuclei isolation.

Flash frozen LAM tissues from WT mice treated with Veh (3 mice), EP (4 mice), and EPR (3 mice) were processed using the Chromium Nuclei Isolation Kit with RNase Inhibitor (10× Genomics, 1000494). Briefly, tissue samples were dissociated and placed in lysis buffer for 14 minutes, then spun down through a Nuclei Isolation Column. After processing with debris removal buffer and wash buffer, nuclei were resuspended in nuclei buffer with RNase inhibitor. Nuclei number was analyzed using Nexcelom Cellometer Auto2000 with AOPI fluorescent staining method.

#### Library preparation, sequencing, and alignment.

10× Genomics multiome library preparation and sequencing was performed at the Northwestern University NUseq facility core. Nuclei first underwent transposition using ATAC enzyme for 1 hour at 37°C. Sixteen thousand transposed nuclei were then loaded into the Chromium Controller (10× Genomics, 120223) on a Chromium Next GEM Chip J (10× Genomics, 1000230) and processed to generate single-cell gel beads in emulsion (GEM) according to the manufacturer’s protocol. Barcoded DNA and cDNA were PCR amplified and subjected to library construction. The snATAC-seq and snRNA-seq libraries were generated using the Chromium Next GEM Single Cell Multiome ATAC + Gene expression kit (10× Genomics, 1000281) and single Index Kit N Set A (10× Genomics, 1000212) according to the manufacturer’s instructions. Amplified cDNA was used for gene expression library preparation using dual Index Kit TT Set A (10× Genomics, 1000215). Quality control for constructed libraries was performed by the Agilent Bioanalyzer High Sensitivity DNA kit (Agilent Technologies, 5067-4626) and the Qubit DNA HS assay kit for qualitative and quantitative analyses, respectively. For snATAC-seq libraries, the multiplexed libraries were pooled and sequenced on an Illumina NovaSeq sequencer with 100 cycles kits using the following read lengths: 50 bp Read1 and 49 bp Read2. For snRNA-seq libraries, the libraries were sequenced on Illumina NovaSeq sequencer with 100 cycles kits using the following read lengths: 28 bp Read 1 for cell barcode and UMI and 90 bp Read 2 for transcript expression. The targeted sequencing depth for snATAC-seq and snRNA-seq was 25,000 and 20,000 reads per cell, respectively. After sequencing, snATAC and snRNA libraries were demultiplexed and aligned with the mouse reference genome (10× Genomics, mm10 2020-A-2.00) using the 10× CellRangerArc 2.0.2 pipeline.

#### Quality control and filtering.

To address ambient RNA common in single-nuclei preparations, each dataset was corrected with SoupX v1.6.2 (https://github.com/constantAmateur/SoupX), which compared raw and filtered expression data to estimate and subtract contaminant transcripts. After decontamination, nuclei with fewer than 500 or more than 10,000 transcripts were excluded. In the ATAC modality, nuclei with fewer than 100 or more than 2,000 fragments were removed, with additional requirements of TSS.enrichment greater than 2 and nucleosome_signal less than 1.

#### Modality-specific normalization, integration, dimension reduction, and clustering.

Filtered RNA and ATAC data were assembled into a single Seurat object per sample. The ATAC assay underwent term frequency–inverse document frequency (TF-IDF) normalization. The latent semantic indexing (LSI) principal components (PCs) 2 through 30 were retained, as component 1 is correlated with the sequencing depth. Gene expression was normalized using SCTransform v0.4.1 (https://github.com/satijalab/sctransform), principal component analysis (PCA) was performed, and the first 30 PCs were retained. Multiple ATAC datasets were integrated by “FindIntegrationAnchors” using reciprocal LSI reduction, followed by “IntegrateEmbeddings” to generate a unified “integrated_lsi” representation. The RNA modality was integrated similarly using reciprocal PCA. Seurat’s weighted nearest neighbor (WNN) pipeline was applied to the integrated RNA and ATAC embeddings for clustering (resolution = 0.8), followed by a UMAP visualization. Cluster identities were annotated by cross-referencing known markers, ATAC accessibility patterns, and relevant literature on cell phenotypes.

#### Differential expression and gene ontology.

Cluster-specific marker genes were identified via “FindAllMarkers” (Wilcoxon’s rank-sum test), filtering for those detected in at least 25% (min.pct = 0.25) of nuclei within a cluster and meeting an adjusted *P*-value threshold (<0.05). Gene Ontology (GO) analyses were conducted with the enrichR (v3.2) GO_Biological_Process_2023 database (https://cran.r-project.org/web/packages/enrichR/index.html Accessed March 1, 2025.). Significantly enriched terms were visualized via EnhancedVolcano (https://github.com/kevinblighe/EnhancedVolcano) and summary plots.

#### Cell-cell communication analysis.

Intercellular communication was inferred using CellChat (v2.1.2) using the full mouse CellChatDB (https://github.com/jinworks/CellChat Accessed March 1, 2025.). For each condition (Veh, EP, EPR), the integrated Seurat object was split and renormalized. Overexpressed genes and interaction communication probabilities were determined using a truncated mean approach (5% trimming) while adjusting for cluster size. Only L-R pairs with adjusted *P* values of less than 0.05 were retained. The 3 CellChat objects were then merged for direct comparisons among conditions, and differentially expressed ligands/receptors were further pinpointed based on 10% minimum detection (thresh.pc = 0.1), fold change greater than 0.05 (thresh.fc = 0.05), and *P* less than 0.05 (thresh.p = 0.05). Interactions were summarized at the pathway level and visualized via bubble plots, heatmaps, and centrality-based analyses. Only pathways with at least 1 significant L-R interaction were included in the pathway-level analysis.

#### ATAC inferred motif activity analysis.

Motif activity was computed from ATAC-seq data using ChromVAR v1.24.0 (https://github.com/GreenleafLab/chromVAR Accessed March 1, 2025.) with motifs from the JASPAR2020 database (https://jaspar.genereg.net/Accessed March 1, 2025.). Differential activity was assessed via Wilcoxon’s rank-sum tests and visualized using violin plots.

### Spatial transcriptomics

Xenium slide processing and Xenium analyzer loading were performed at the Northwestern University NUSeq facility core. LAM sections of 5 μm thickness were placed on the capture area of a Xenium slide, followed by 3-hour incubation at 42°C and overnight drying. Xenium analyzer loading was carried out using the slide and sample prep reagent kit (Xenium, 1000460), decoding consumables (Xenium, 1000487), and decoding reagent (Xenium, 1000461). Slides were first deparaffinized and de-crosslinked according to manufacturer’s protocol (Xenium, CG000580). Then, custom probes with predesigned panel genes were hybridized overnight, followed by probe ligation and amplification, according to the manufacturer’s protocol (CG000582 for Xenium in situ gene expression; CG000749 for Xenium in situ gene expression with cell segmentation; and CG000760 for Xenium Prime in situ gene expression with cell segmentation). Autofluorescence quenching and nuclei staining were also performed before imaging and decoding signals on the Xenium Analyzer.

The Xenium data were initially filtered to retain high-quality cells by excluding the cells that had fewer than 50 or more than 2,000 reads, or if they expressed fewer than 50 or more than 750 genes. Additionally, cells with a mitochondrial transcript percentage exceeding 10% were removed. The reads were then normalized with SCTransform and PCA was performed to compute the top 30 PCs. The samples were integrated using canonical correlation analysis (CCAIntegration), followed by clustering and UMAP visualization. The clusters were annotated using the resolution of 0.3 for cell-type identification.

### RNAscope assay

Fluorescent in situ mRNA detection for transcripts was performed on human LAM muscle samples using the RNAscope Multiplex Fluorescent Reagent Kit v2 (ACD Bio, 323100) with pretreatment reagents (ACD Bio, 322381). Briefly, 5-μm-thick formalin-fixed paraffin-embedded tissue sections were pretreated with heat and protease before hybridization. Slides were processed according to the manufacturer’s instructions. Tissue sections were hybridized with RNAscope target probes, with probes to the *DapB* bacterial gene (ACD Bio, 310043) and the endogenous human *UBC* gene (ACD Bio, 310041) used as negative and positive controls, respectively. Probes included Hs-FBXO32 (ACD, 521671), Hs-TRIM63 (ACD, 532291), Hs-TGFBR2 (ACD, 407941), Hs-FBN1 (ACD, 482471), Hs-WT1 (ACD, 415581), Hs-PGR-O1 (ACD, 435961), and Hs-TGFB2 (ACD, 489241). Positive mRNA expression was demonstrated by punctate fluorescent signal present within the cytoplasm or nucleus. Probe signal was quantified using ImageJ.

### Statistics

Analysis of sequencing data was performed as described above. For all other comparisons, means and SEM were determined for measured parameters. A 2-tailed *t* test was used for experiments with 2 groups, and ANOVA was used for experiments with more than 2 groups. Post hoc multiple pairwise comparisons were corrected using the Dunn-Bonferroni method. Spearman’s correlation coefficient was used for correlation analyses. A *P* value of less than 0.05 was considered significant. Nonsequencing data analyses were carried out using GraphPad Prism.

### Study approval

All experimental protocols involving animals were approved by the Institutional Animal Care and Use Committee at Northwestern University. Human studies were approved by the Institutional Review Board of Northwestern University and the University of Texas Health Science Center at Houston. Written informed consent was obtained from all patients before hernia surgery performed at the University of Texas Health Science Center at Houston (STU00208860).

### Data availability

Values for all data points in graphs are reported in the Supporting Data Values file. The raw sequencing data and processed files supporting this study have been deposited in the NCBI Gene Expression Omnibus (GEO). Single-nuclei multiomic sequencing data can be found under accession number GSE288662. Xenium spatial transcriptomics data can be found under accession number GSE288663.

## Author contributions

TY and MZ wrote the original draft of the manuscript. TY, MZ, TP, NP, JSC, EB, MK, and DJE performed experiments. TY, MZ, and YD analyzed data and generated figures. JJS contributed to patient recruitment and specimen collection. SEB and HZ conceived and supervised the study. All authors contributed to methodology and reviewing and editing the manuscript.

## Supplementary Material

Supplemental data

Unedited blot and gel images

Supporting data values

## Version 1 06/12/2025

In-Press Preview

## Version 2 07/22/2025

Electronic publication
