# Supplementary material for: Role of progesterone action in inguinal hernia formation via skeletal muscle fibrosis and atrophy
Source: JCI Insight. 2025 Jun 12;10(14):e193208. doi: 10.1172/jci.insight.193208 (PMC12288974; doi:10.1172/jci.insight.193208)
Supplement: Supplemental data [file jciinsight-10-193208-s199.pdf]

Supplementary Files

Role of Progesterone Action in Inguinal Hernia Formation via Skeletal Muscle Fibrosis and Atrophy

Authors
Tianming You[1], Mehrdad Zandigohar[2], Tanvi Potluri[1], Natalie Piehl[1], John Coon V[1], Elizabeth Baker[1], Maya Kafali[1], Yang Dai[2], Jonah J. Stulberg[3], David J. Escobar[4], Richard L. Lieber[5-7], Hong Zhao[1†]* and Serdar E. Bulun[1†]*

Affiliations
[1]Department of Obstetrics & Gynecology, Feinberg School of Medicine, Northwestern University, Chicago, USA
[2]Department of Biomedical Engineering, University of Illinois Chicago, Chicago, USA
[3]Department of Surgery, McGovern Medical School at the University of Texas Health Sciences Center, Houston, USA
[4]Department of Pathology, Feinberg School of Medicine, Northwestern University, Chicago, USA
[5]Departments of Physical Medicine and Rehabilitation and Biomedical Engineering, Northwestern University, Chicago, USA
[6]Research Service, Hines VA Medical Center, Maywood, IL, USA
[7]Shirley Ryan AbilityLab, Chicago, USA
[†]These authors jointly supervised this work

The authors have declared that no conflicts of interest exist.

*Corresponding Authors

Serdar E. Bulun, M.D.
Department of Obstetrics & Gynecology
Feinberg School of Medicine
Northwestern University, Chicago, 60611, USA
Phone: +1 312.472.3980
Email: s-bulun@northwestern.edu

Hong Zhao, M.D., Ph.D.
Department of Obstetrics & Gynecology
Feinberg School of Medicine
Northwestern University, Chicago, 60611, USA
Phone: +1 312.503.0780
Email: h-zhao@northwestern.edu

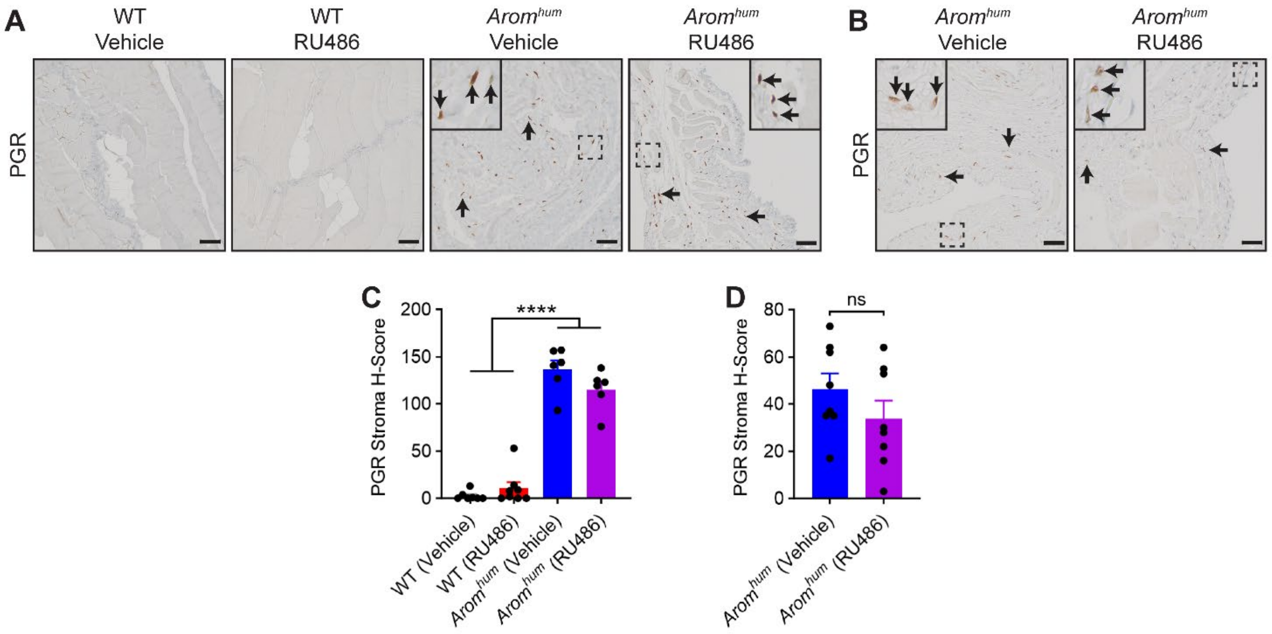

Supplementary Figure S1. Expression of PGR in the LAM of RU486-treated WT and *Arom^{hum}* mice. (A-B) Representative PGR immunohistochemistry staining images **(A)** and quantification **(B)** of stromal PGR expression in LAM tissues of WT and *Arom*^{hum} mice after 12-week RU486 preventive treatment (n = 6-8/group, mean ± S.E.M., two-way ANOVA). **(C-D)** Representative PGR immunohistochemistry staining images **(C)** and quantification **(D)** of stromal PGR expression in LAM tissues after 12-week RU486 treatment of established hernias in *Arom*^{hum} mice. (n = 8/group, mean ± S.E.M., t-test). Scale bar, 50 µm. ****$p < 0.0001$.

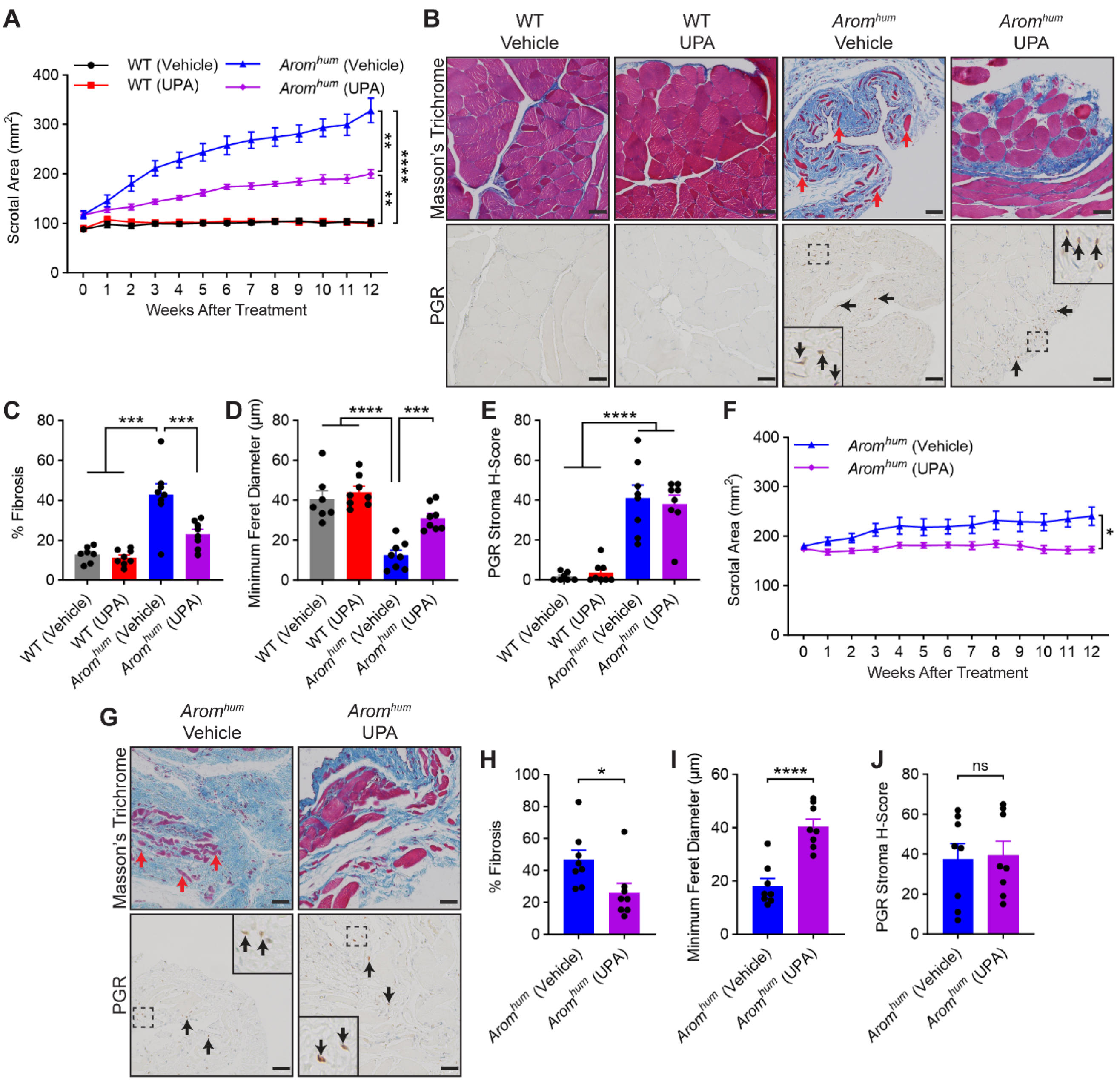

Supplementary Figure S2. Ulipristal acetate (UPA) treatment prevents hernia development and delays further hernia growth in *Arom*^{hum} mice. (A) Scrotal/hernia area measurements of WT and *Arom*^{hum} mice treated with UPA for hernia prevention (n = 10-11/group, mean ± S.E.M., repeated measures ANOVA). **(B)** Representative images of LAM Masson's trichrome staining and immunohistochemistry staining for PGR in WT and *Arom*^{hum} mice after 12-week UPA preventive treatment. Red arrows show atrophying myofibers in herniated LAM tissue in vehicle-treated *Arom*^{hum} mice. **(C-E)** Quantification of **(C)** fibrotic area, **(D)** minimum Feret diameter, and **(E)** stromal PGR expression in LAM tissues after 12-week UPA preventive treatment (n = 7-8/group, mean ± S.E.M., two-way ANOVA). **(F)** Scrotal/hernia area measurements of *Arom*^{hum} mice with established hernias treated with UPA (n = 11-12/group, mean ± S.E.M., multiple t-test). **(G)** Representative images of LAM Masson's trichrome staining

and immunohistochemistry staining for PGR after 12 weeks of UPA treatment of established hernias in *Arom*^{hum} mice. Red arrows show atrophying myofibers in herniated LAM tissue in vehicle-treated *Arom*^{hum} mice. **(H-J)** Quantification of **(H)** fibrotic area, **(I)** minimum Feret diameter, and **(J)** stromal PGR expression in LAM tissues after 12 weeks of UPA treatment of established hernias in *Arom*^{hum} mice (n = 8/group, mean ± S.E.M., t-test). Scale bar, 50 µm. *p < 0.05, **p < 0.01, ***p < 0.001, ****p < 0.0001.

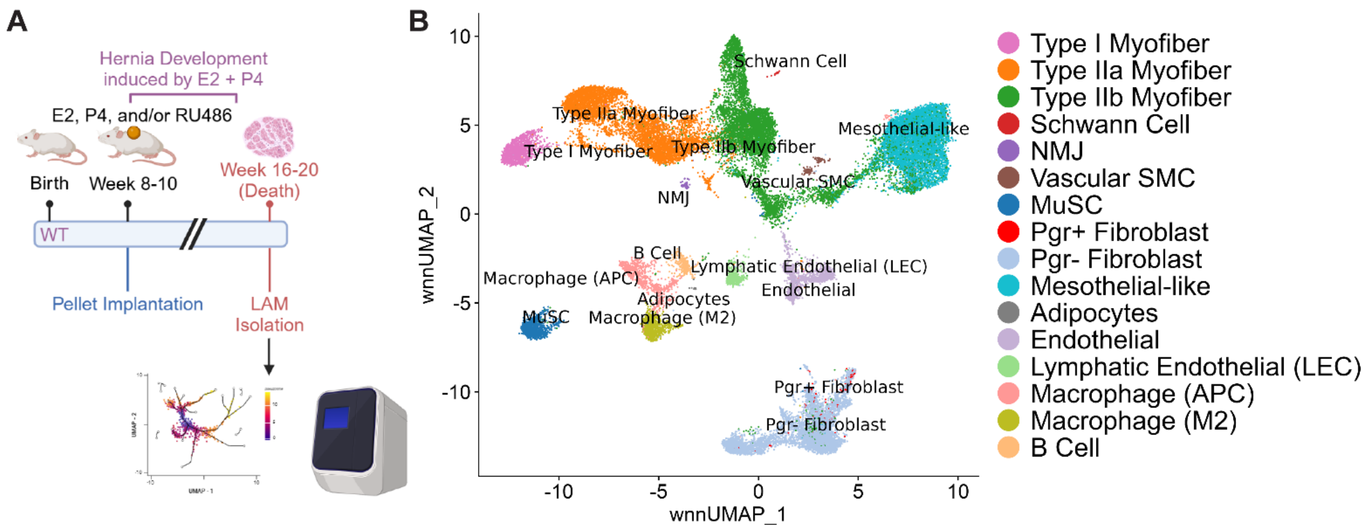

Supplementary Figure S3. Combined cell type composition from LAM tissue of WT mice treated with E2, P4, and/or RU486. (A) Schematic for the treatment of WT mice with E2, P4, and/or RU486 for LAM single-nuclei isolation. **(B)** UMAP plot of all Vehicle (Veh), E2 + P4 (EP), and E2 + P4 + RU486 (EPR) LAM nuclei combined after weighted nearest neighbor (wnn) integration, separated by cell type. A total of 16 distinct cell types were identified and grouped based on canonical marker expression.

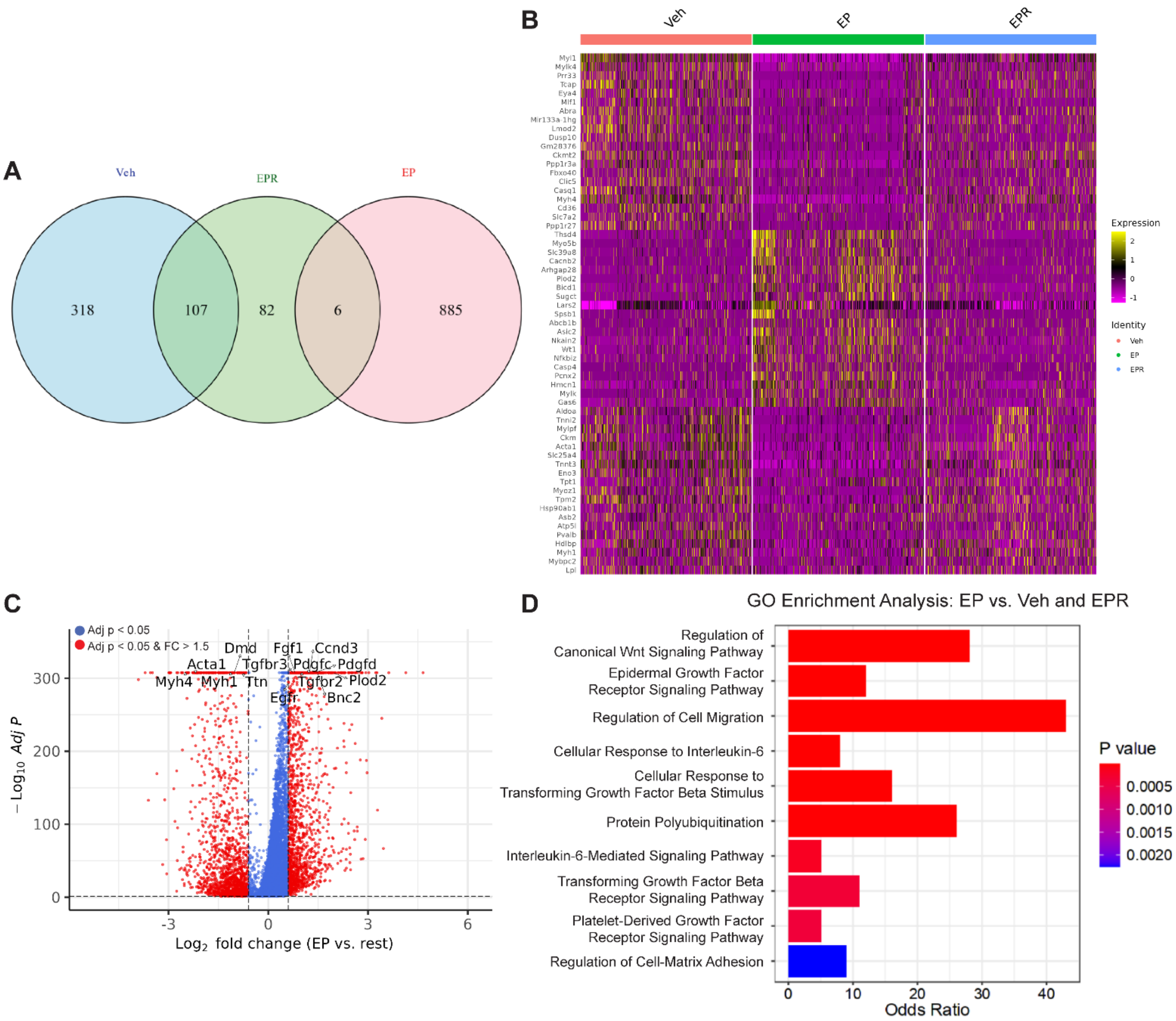

Supplementary Figure S4. Increased inflammatory and fibrotic signaling in bulk transcriptomics of LAM tissue from WT mice treated with E2, P4, and/or RU486. (A) Venn diagram showing overlap of upregulated genes in the entire LAM of WT mice treated with vehicle (Veh), E2 + P4 (EP), and E2 + P4 + RU486 (EPR) (fold change > 1.5, Wilcoxon rank-sum test, $p < 0.05$). **(B)** Heatmap of top 20 upregulated genes in entire Veh, EP, and EPR LAM. **(C)** Volcano plot showing upregulated and downregulated genes in the entire EP LAM relative to both the entire Veh LAM and EPR LAM (fold change > 1.5, Wilcoxon rank-sum test, $p < 0.05$). **(D)** Gene Ontology (GO) processes enriched in the entire EP LAM, represented by odds ratio.

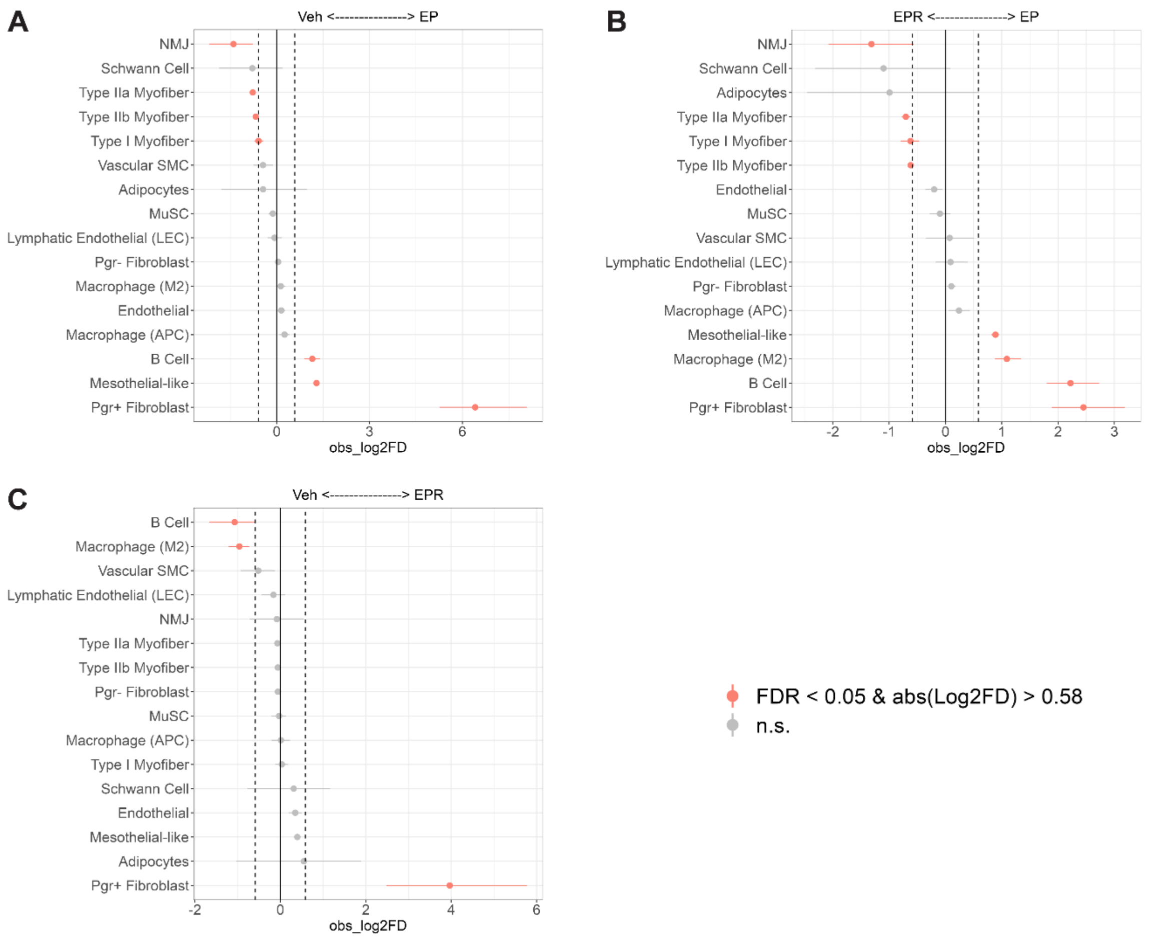

Supplementary Figure S5. Differences in cell type proportion between Vehicle (Veh), E2 + P4 (EP), and E2 + P4 + RU486 (EPR) LAM. (A-C) Forest plot depicting the difference in each cell type's proportion in **(A)** EP vs. Veh, **(B)** EP vs. EPR, and **(C)** EPR vs. Veh LAM. A red dot indicates a statistically significant difference in proportion of that cell type between the two treatment groups. Horizontal lines indicate the range of the 95% confidence interval (fold change > 1.5, permutation test with FDR correction, $p < 0.05$).

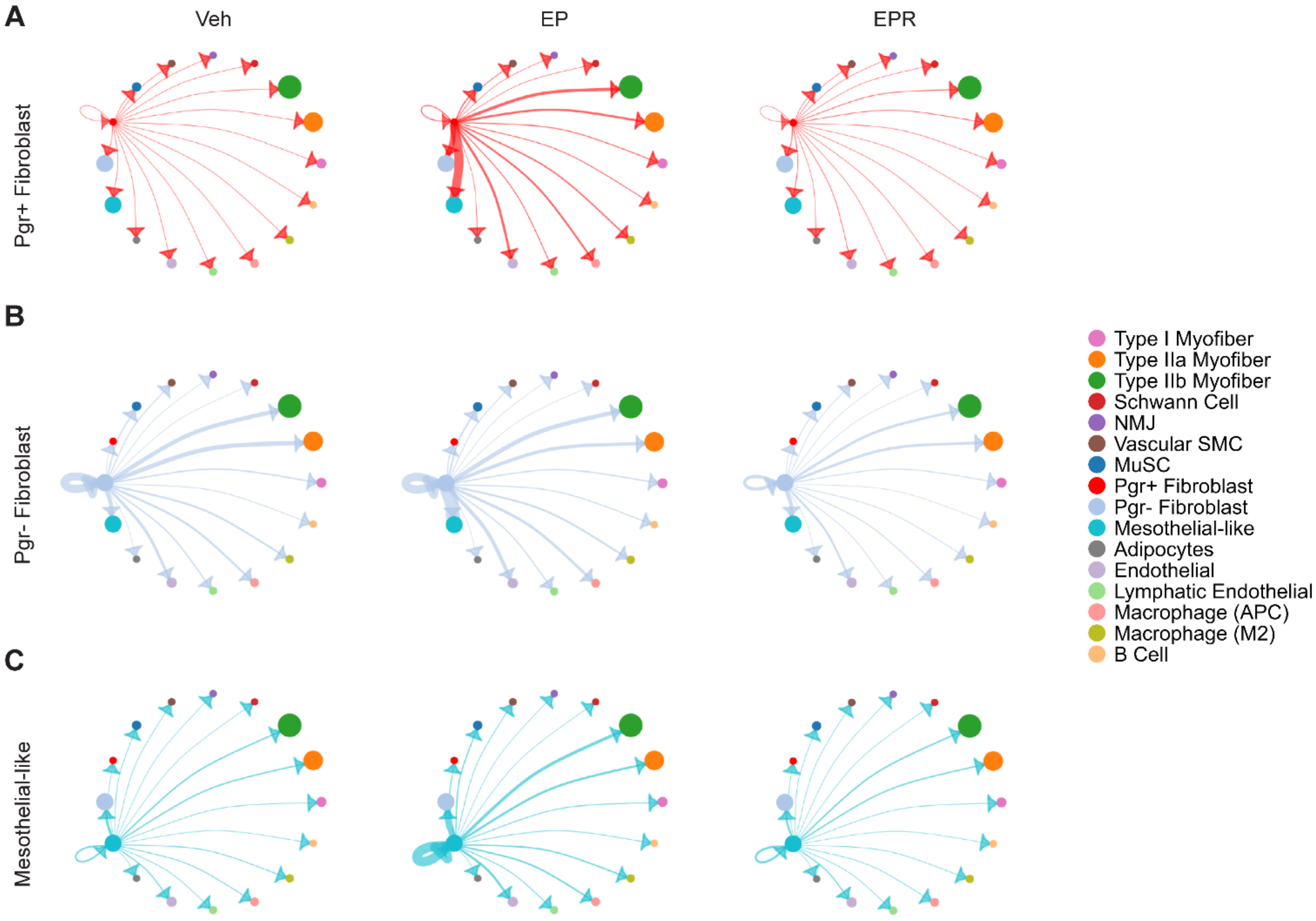

Supplementary Figure S6. Outgoing communication networks from *Pgr*$^{+}$ fibroblasts, *Pgr*$^{-}$ fibroblasts, and mesothelial-like cells to other LAM cell types. (A-C) Visualization of cell-cell communication using CellChat, comparing communication networks between Vehicle (Veh), E2 + P4 (EP), and E2 + P4 + RU486 (EPR) LAM. Circle plots with **(A)** *Pgr*$^{+}$ fibroblasts, **(B)** *Pgr*$^{-}$ fibroblasts, and **(C)** mesothelial-like cells as the central outgoing nodes. Interactions between pairs of cell types are depicted by a line connecting the two cell types, with line thickness indicating the strength of the interaction.

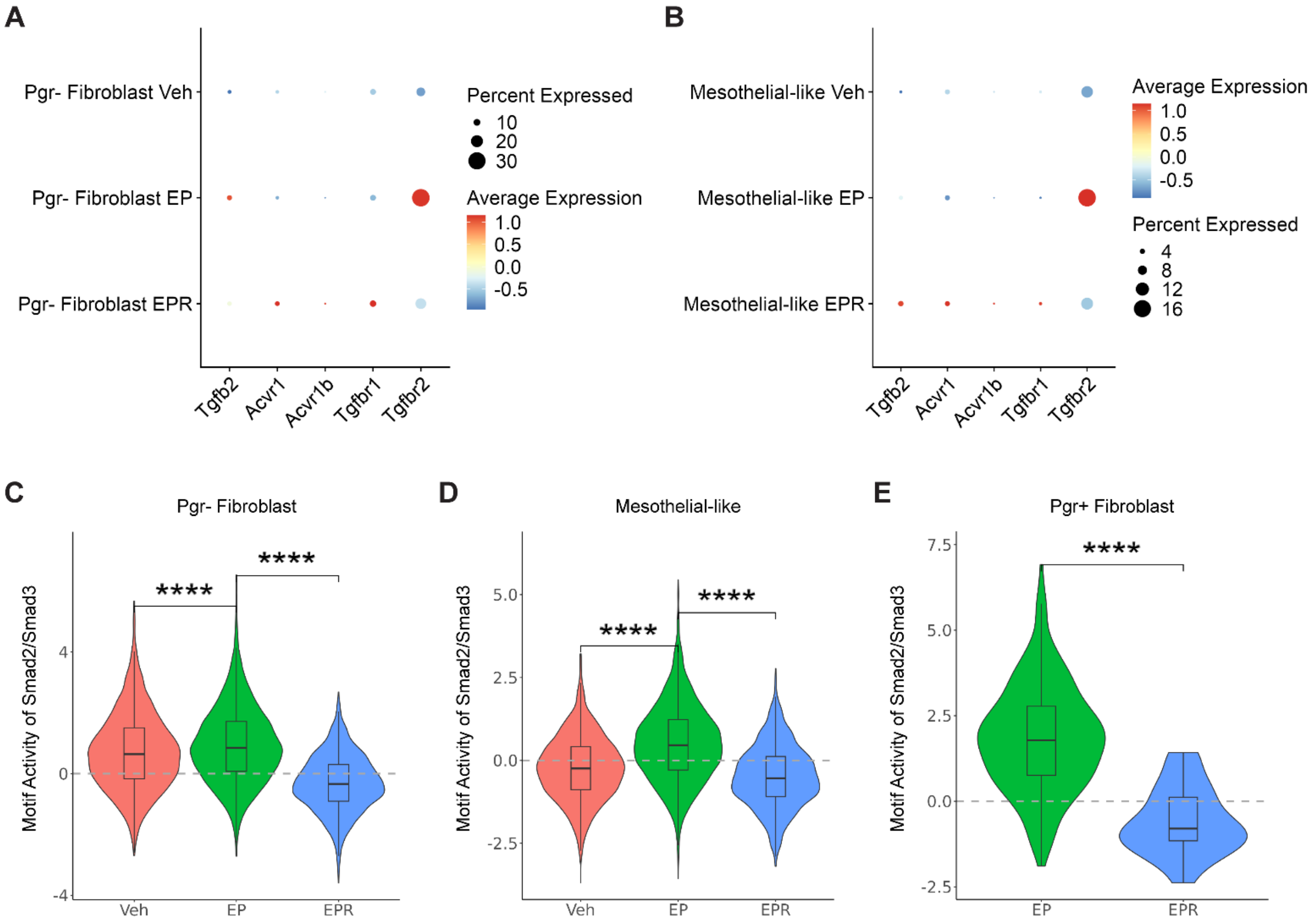

Supplementary Figure S7. Expression of TGFβ pathway ligands and receptors in *Pgr*$^-$ fibroblasts and mesothelial-like cells. (A-B) Comparison of *Tgfb2*, *Acvr1*, *Acvr1b*, *Tgfbr1*, and *Tgfbr2* expression in Vehicle (Veh), E2 + P4 (EP), and E2 + P4 + RU486 (EPR) LAM for **(A)** *Pgr*$^-$ fibroblasts and **(B)** mesothelial-like cells. Size of dots corresponds to frequency of expression within a treatment group. Color of dots corresponds to average expression level within the treatment group. **(C-D)** Comparison of inferred motif activity of Smad2/Smad3 (downstream transcription factors in the TGFβ pathway) in Veh, EP, and EPR LAM for **(C)** *Pgr*$^-$ fibroblasts and **(D)** mesothelial-like cells. **(E)** Inferred motif activity of Smad2/Smad3 in *Pgr*$^+$ fibroblasts. Wilcoxon rank-sum test, ****$p < 0.0001$.

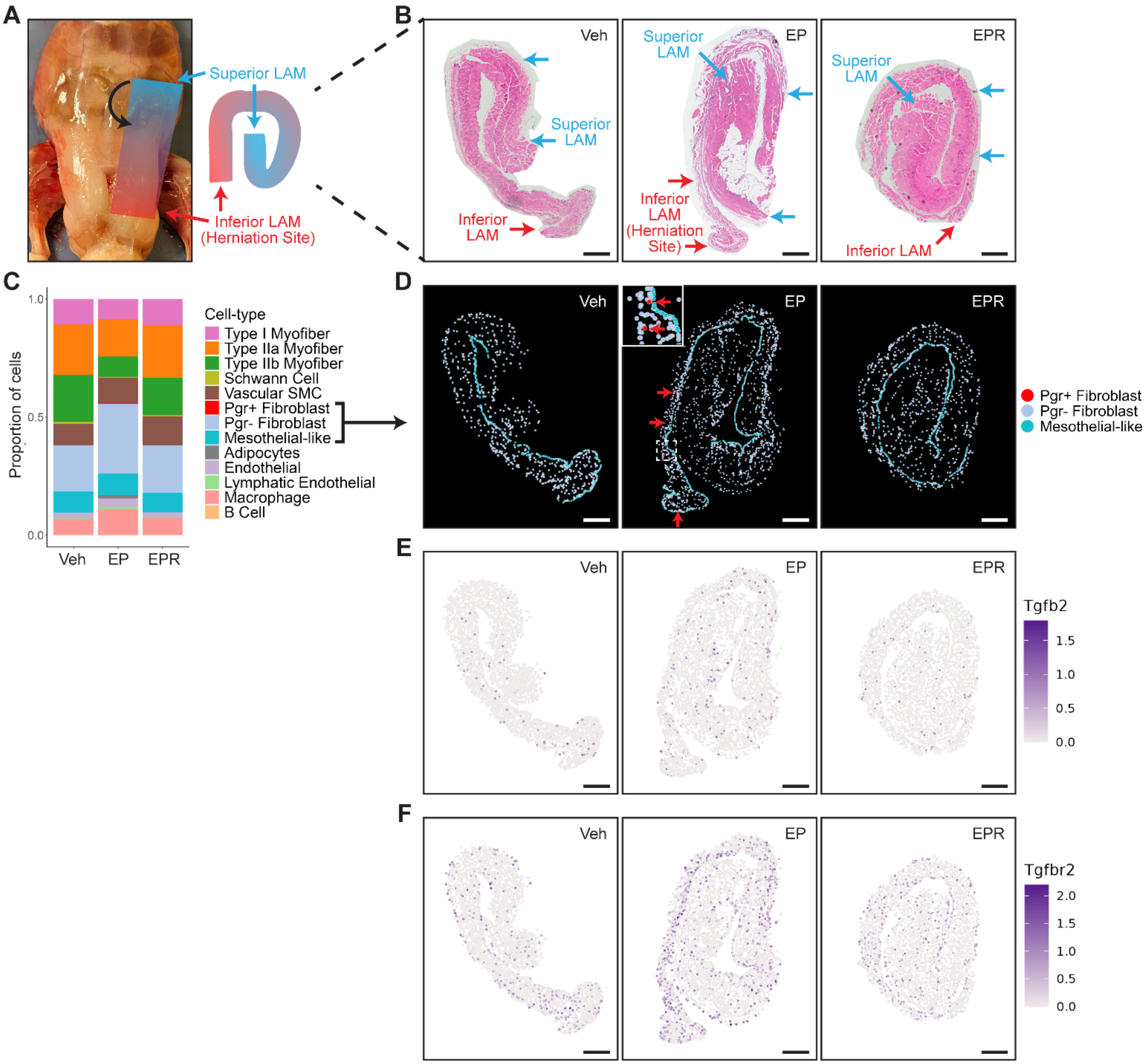

Supplementary Figure S8. Expression and spatial distribution of *Tgfb2* and *Tgfbr2* in the LAM of mice with E2/P4-induced hernias. (A) Schematic depicting the isolation, rolling up, and final orientation of LAM tissue for Xenium spatial transcriptomics. **(B)** Hematoxylin and eosin staining of LAM tissue from WT mice treated with vehicle (Veh), E2 + P4 (EP), and E2 + P4 + RU486 (EPR). Superior (cyan arrows) and inferior (red arrows) portions of LAM are highlighted. **(C)** Cell-type compositional makeup and **(D)** spatial distribution of *Pgr*$^{+}$ fibroblasts, *Pgr*$^{-}$ fibroblasts, and mesothelial-like cells in Veh, EP, and EPR LAM. *Pgr*$^{+}$ fibroblasts in EP LAM are highlighted (red arrows). **(E-F)** Spatially resolved gene expression of **(E)** *Tgfb2* and **(F)** *Tgfbr2* in Veh, EP, and EPR LAM. Scale bar, 500 μm.

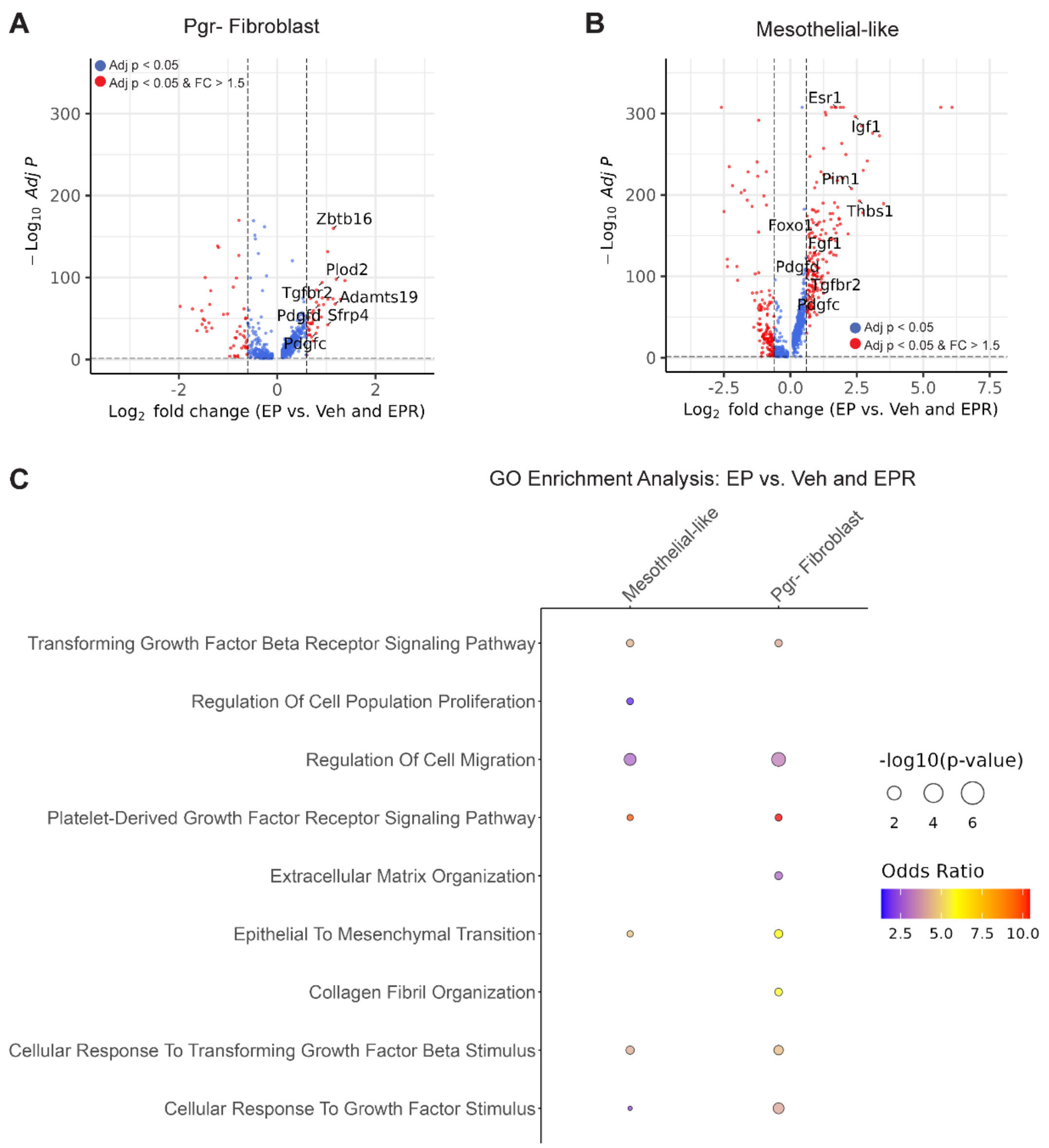

Supplementary Figure S9. DEG analysis of *Pgr*$^{-}$ fibroblasts and mesothelial-like cells from LAM of mice with E2/P4-induced hernias. (A-B) Volcano plots showing upregulated and downregulated genes in **(A)** *Pgr*$^{-}$ fibroblasts and **(B)** mesothelial-like cells from E2 + P4 (EP) LAM compared to Vehicle (Veh) and E2 + P4 + RU486 (EPR) LAM (fold change > 1.5, Wilcoxon rank-sum test, $p < 0.05$). **(C)** Dot plot showing Gene Ontology (GO) processes enriched in EP LAM *Pgr*$^{-}$ fibroblasts and mesothelial-like cells, represented by odds ratio. n = 3-4/group.

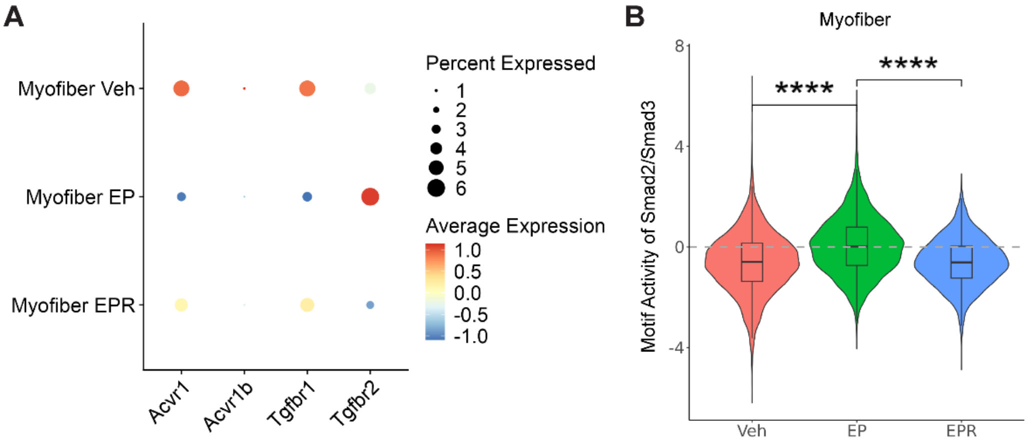

Supplementary Figure S10. Expression of TGFβ pathway receptors in myofibers. (A) Relative expression of *Acvr1*, *Acvr1b*, *Tgfbr1*, and *Tgfbr2* for all myofiber types in Vehicle (Veh), E2 + P4 (EP), and E2 + P4 + RU486 (EPR) LAM. Size of dots corresponds to frequency of expression within a treatment group. Color of dots corresponds to average expression level within the treatment group. **(B)** Comparison of inferred motif activity of Smad2/Smad3 (downstream transcription factors in the TGFβ pathway) for all myofiber types in Veh, EP, and EPR LAM. Wilcoxon rank-sum test, ****$p < 0.0001$.

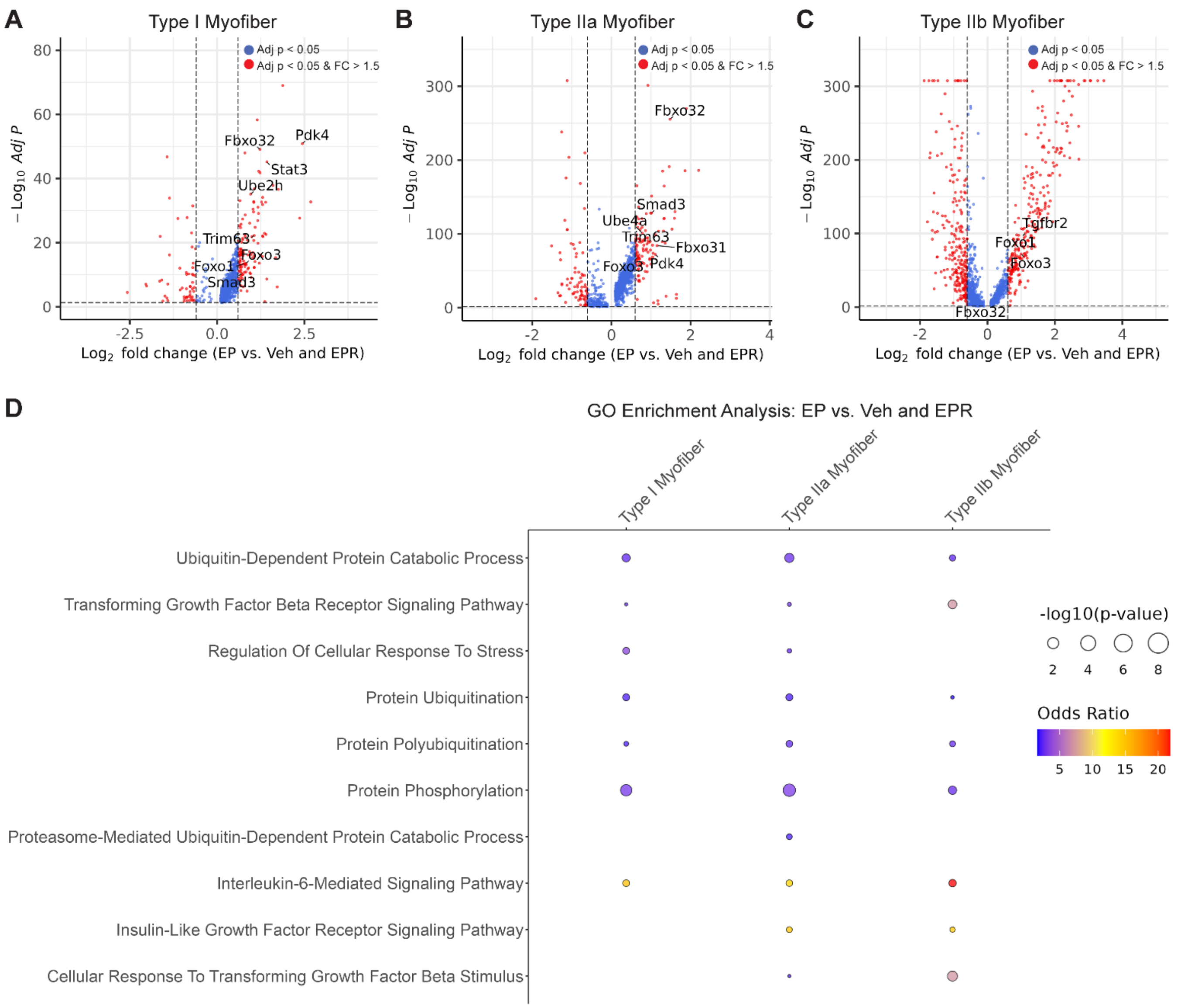

Supplementary Figure S11. DEG analysis of myofibers from LAM of mice with E2/P4-induced hernias. (A-C) Volcano plots showing upregulated and downregulated genes of **(A)** type I, **(B)** type IIa, and **(C)** type IIb myofibers in E2 + P4 (EP) LAM compared to Vehicle (Veh) and E2 + P4 + RU486 (EPR) LAM (fold change > 1.5, Wilcoxon rank-sum test, $p < 0.05$). **(D)** Dot plot showing Gene Ontology (GO) processes enriched in EP LAM type I, type IIa, and type IIb myofibers, represented by odds ratio. n = 3-4/group.

Materials and Methods

Fibroblast Cell Culture

Fibroblast Isolation: Fibroblasts were isolated as previously described (1). Briefly, mouse LAM tissues were harvested and placed in wash media (Hyclone Ham's F-10 nutrient mixture with 1mM L-glutamate [GE Life Sciences], 10% horse serum [Life Technologies], and penicillin-streptomycin [Gibco]) on ice. LAM tissues were minced into a slurry and incubated in muscle dissociation buffer (wash media with 1000U/mL collagenase II [Worthington Biochemical]) at 37°C with 70 rpm agitation for 1.5 hours. The cells were then washed and resuspended in fibroblast growth media (Ham's F-12 media with 10% FBS [Gibco], penicillin-streptomycin [Gibco], and plasmocin prophylactic [InvivoGen]) and grown on 0.15% gelatin-coated tissue culture plates. After waiting 1 hour to allow for LAM fibroblasts to adhere to the plate, the supernatant containing other cell types was removed. Cells were grown to 90% confluence before passaging.

Fibroblast Treatments: After one passage, primary fibroblasts were grown to ~60-70% confluency, then starved overnight (16 hours) in phenol-red free, serum-free media (Ham's F-12 without phenol-red with penicillin-streptomycin [Gibco] and plasmocin prophylactic [InvivoGen]). E2 antagonists (Fulvestrant, 100 nM in DMSO) and P4 antagonists (RU486, 1 μM in ethanol; UPA, 1μM in ethanol; ZK299, 1 μM in ethanol) were added 2 hours before E2 (10 nM in ethanol) and R5020 (a synthetic version of P4, 100 nM in ethanol) for all experiments. During treatment, 0.1% charcoal-stripped FBS (Gibco) was added to ensure cell survival during longer incubation times (24-48 hours).

Immunoblot: Primary fibroblasts were cultured, starved, and treated as outlined in *Fibroblast Treatments*. To extract protein, fibroblasts were incubated for 1.5 hours at 4°C in Pierce™ IP Lysis Buffer (Thermo Fisher Scientific, #87787) with 1:100 Halt™ Protease Inhibitor Cocktail (Thermo Fisher Scientific, #78430). Protein concentrations in the lysate were measured via BCA Assay (Thermo Fisher Scientific, #A55864). Protein samples were prepared with LDS Buffer containing 10% BME, separated via SDS-PAGE, and subsequently transferred to polyvinylidene difluoride membranes (Thermo Fisher Scientific). Membranes were blocked in 5% nonfat milk dissolved in Tris-buffered saline containing 0.1% Tween-20 (Sigma-Aldrich, #9005-64-5, TBST), incubated overnight at 4°C with primary antibody, washed with TBST, and incubated with horseradish peroxidase (HRP)-conjugated secondary antibody at room temperature. Immunoreactivity was detected with Immobilon Crescendo Western HRP substrate (Millipore, #WBLUR0100). Primary antibodies used included: mouse anti-PGR monoclonal antibody (Invitrogen, #MA5-12658; discontinued), rabbit anti-PGR polyclonal antibody (Abclonal, #A0321), and HRP-conjugated mouse anti-β-actin monoclonal antibody (Proteintech, #60008). Secondary antibodies used included: HRP-conjugated goat anti-rabbit IgG (Cell Signaling, #7074) and HRP-conjugated horse anti-mouse IgG (Cell Signaling, #7076).

EdU Assay: The contents and concentrations of all culture media, hormones, and drugs used were the same as in *Fibroblast Treatments*. Primary fibroblasts were transferred to 96-well plate and seeded at a density of 10,000 cells per well. Fibroblasts were given time to attach (~24 hours), then starved overnight (16 hours) before being treated with E2, R5020, RU486, UPA, and/or ZK299 in starvation media with 0.1% charcoal-stripped FBS added. After incubation for 24 hours, the EdU Assay was performed using the Click-iT™ EdU Proliferation Assay for

Microplates (Invitrogen, #C10499). Fibroblasts were incubated with 10µM EdU for 3.5 hours before fixation and click labeling with Amplex™ UltraRed for 15 minutes. Sample wells were read on a microplate reader using an excitation wavelength of 568nm and an emission wavelength of 585nm.

siRNA Knockdown Treatments: The contents and concentrations of all culture media, hormones, and drugs used were the same as in *Fibroblast Treatments*. Fibroblasts were given time to attach (~24 hours), then starved overnight (16 hours) before being treated with E2, R5020, and/or siRNA in starvation media with 0.1% charcoal-stripped FBS added. siRNA treatment involved addition of 75nM *Pgr* siRNA (Dharmacon, L-057577-01-0005) or 75nM non-targeting siRNA (Dharmacon, D-001810-10-05) and 1:200 DharmaFECT 1 transfection reagent (Dharmacon, T-2001-02).

References

1. Judson RN, Low M, Eisner C, and Rossi FM. Isolation, Culture, and Differentiation of Fibro/Adipogenic Progenitors (FAPs) from Skeletal Muscle. *Methods Mol Biol.* 2017;1668:93-103.
