# Supplementary material for: Role of progesterone action in inguinal hernia formation via skeletal muscle fibrosis and atrophy
Source: JCI Insight. 2025 Jun 12;10(14):e193208. doi: 10.1172/jci.insight.193208 (PMC12288974; doi:10.1172/jci.insight.193208)
Supplement: Unedited blot and gel images [file jciinsight-10-193208-s200.pdf]

Full unedited gel for Figure 1C

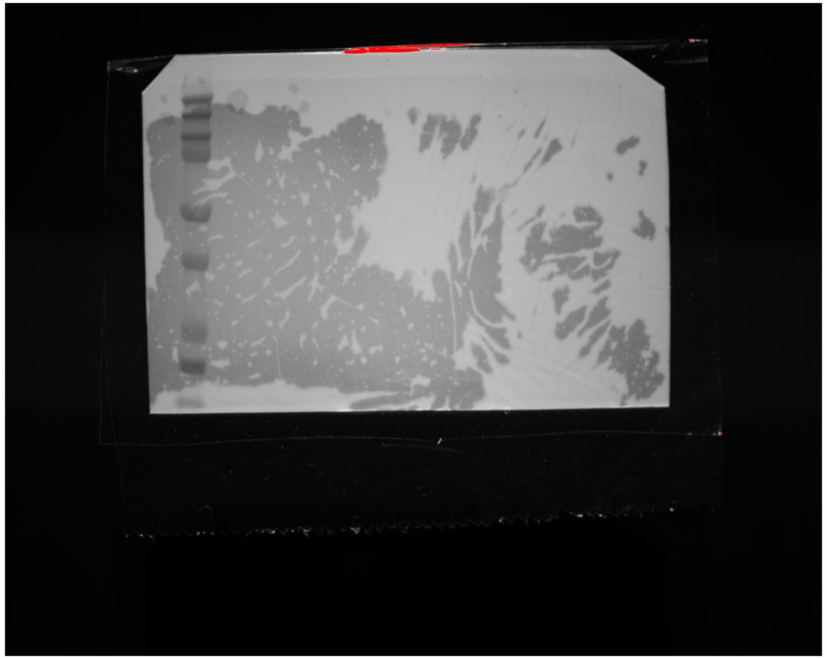

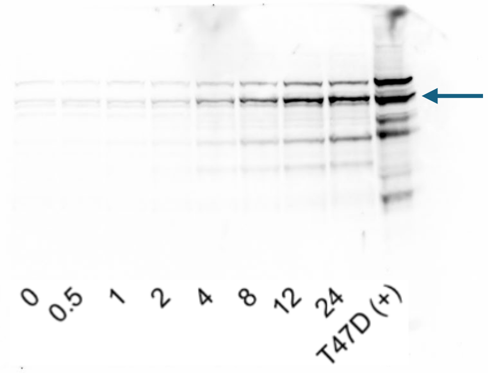

Anti-PGR Ab (Invitrogen, MA5-12658)

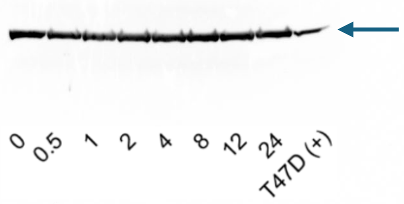

Anti-β-actin Ab (Proteintech, 60008)

Full unedited gel for Figure 1D

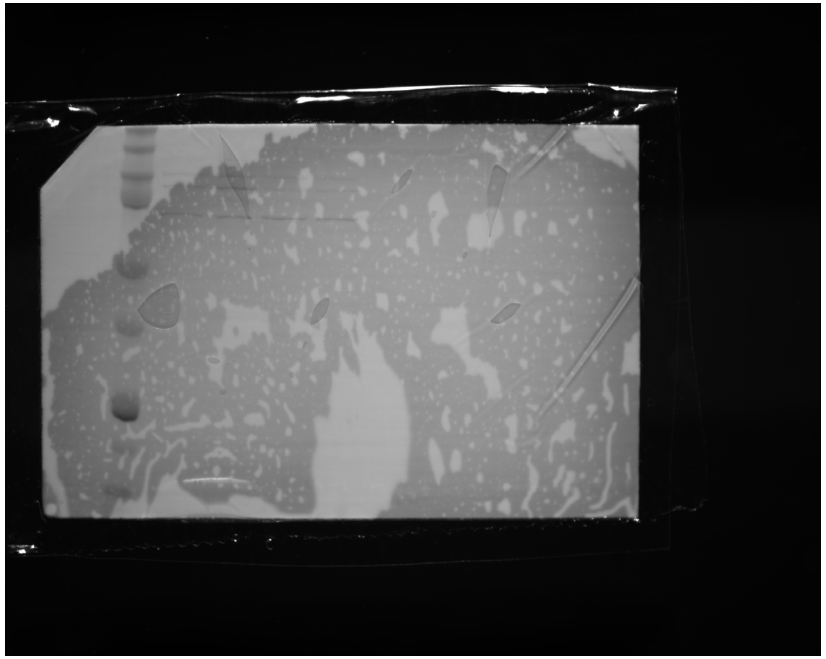

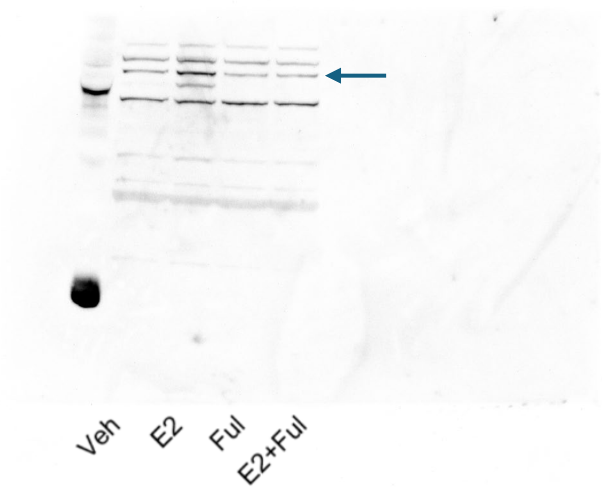

Anti-PGR Ab (Abclonal, A0321)

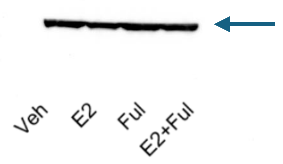

Anti-β-actin Ab (Proteintech, 60008)

Full unedited gel for Figure 1F

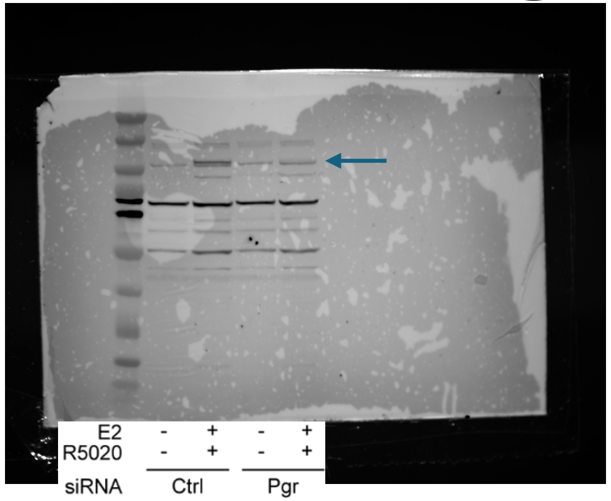

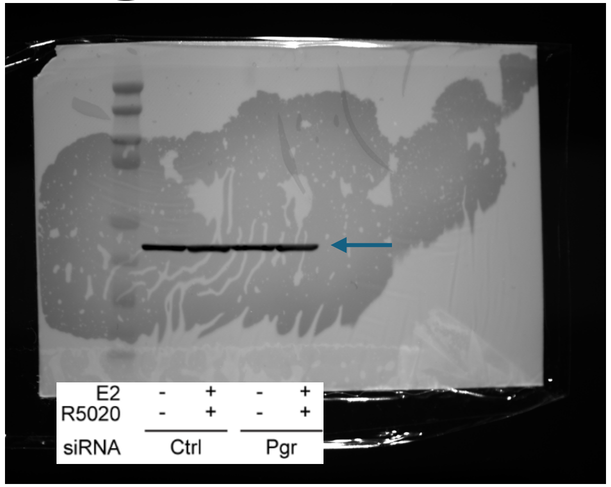

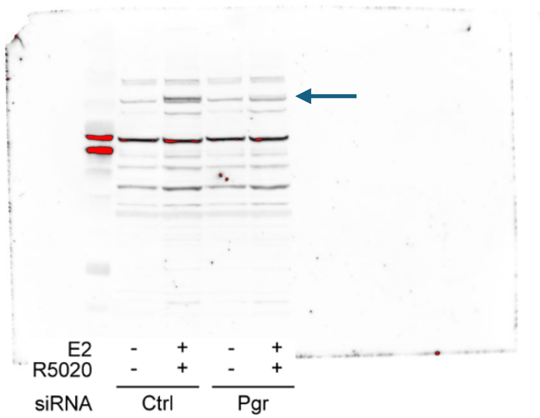

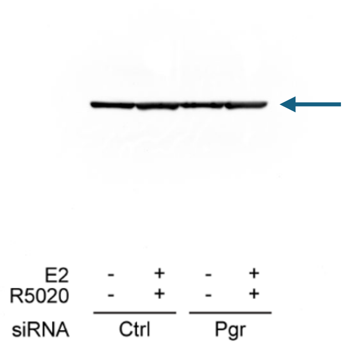

Anti-PGR Ab (Abclonal, A0321)

Anti-β-actin Ab (Proteintech, 60008)
